# Effectiveness of acupuncture and related therapies for post-stroke insomnia: A systematic review and Bayesian network analysis

**DOI:** 10.1097/MD.0000000000050081

**Published:** 2026-08-07

**Authors:** Jie Tang, Huizhong Tan, Liangyi Xiao, Shanshan Zeng, Shiliang Wang, Qian Liu, Yao Xie, Junlin Jiang, Le Xie, Dahua Wu

**Affiliations:** a Graduate School, Hunan University of Chinese Medicine, Changsha, China; b Department of Neurology, Hunan Provincial Hospital of Integrated Traditional Chinese and Western Medicine (The Affiliated Hospital of Hunan Academy of Traditional Chinese Medicine), Changsha, China; c Department of Acupuncture Rehabilitation, Changsha Hospital of Traditional Chinese Medicine (Changsha No. 8 Hospital), Changsha, China.

**Keywords:** acupuncture, insomnia, network meta-analysis, randomized controlled trials, stroke

## Abstract

**Background:**

Poststroke insomnia is often associated with poor recovery. Acupuncture (Acu) and related therapies offer have notable advantages as effective and safe treatment for poststroke insomnia. This systematic review evaluated the therapeutic effect of various Acu interventions on poststroke insomnia.

**Methods:**

Two researchers independently screened literature and extracted data from 8 databases for randomized controlled trials of Acu and related therapies for poststroke insomnia. The risk of bias was assessed using the “bias risk assessment tool” recommended by the Cochrane Library. R version 4.4.1 and Stata 18.0 were used to conduct a network meta-analysis of relevant outcomes.

**Results:**

A total of 107 articles were included, involving 8062 patients and 14 Acu related therapies. Network meta-analysis revealed that in terms of Pittsburgh Sleep Quality Index total score, Acu related therapies were superior to western medicine and conventional rehabilitation therapy. The combined therapy, Acu + auriculotherapy + electroacupuncture had the best outcomes. Acu alone, Acu + auriculotherapy + point embedding and Acu + western medicine had better effects on prolonging sleep duration and shortening sleep latency. For subjective sleep quality, point embedding achieved the best outcomes. Warm acupuncture demonstrated the best results in improving daily functional dysfunction, while Acu ranked the as the best in reducing the degree of sleep disorder.

**Conclusion:**

Acu and related therapies have a positive effect in ameliorating poststroke insomnia, with the combined therapy having a more significant effect. However, different Acu regimens should be considered for poststroke insomnia patients with different clinical manifestations. More standardized clinical trials are needed to provide higher quality evidence and optimal treatment regimen.

## 1. Introduction

An aging population and changes in lifestyle have contributed to an increased exposure to risk factors such as hypertension leading to a rise in and cerebrovascular diseases.^[1]^ Stroke has become the second leading cause of death and the third leading cause of disability globally.^[2,3]^ As the number of stroke survivors increases, the sequelae of stroke have emerged as a significant issue affecting patients’ physical and mental health of patients. These patients often require sufficient and long-term rehabilitation care, which imposes a substantial economic burden.^[4]^

Sleep disorders are a common in stroke cases and is often associated with poor recovery. Insomnia is one of the most common mental health problems, with an incidence of about 30% to 38% in the first year after stroke, which increases with age and follow-up time.^[5]^ Evidence indicates a bidirectional relationship between stroke and insomnia. Stroke may be a predispose individuals to insomnia, and insomnia can worsen stroke prognosis of and increase the recurrence risk.^[6–8]^ Benzodiazepines are commonly used for poststroke insomnia, but may cause breathing-related sleep disorders, movement disorder recurrence,^[9]^ addiction, and drug resistance, hindering recovery. Acupuncture (Acu) is a simple safe and effective therapy and a key component of traditional Chinese medicine. It has unique advantages in the treatment of poststroke insomnia, and its curative effects have been verified by relevant studies.^[10]^ However, optimal Acu and related therapies are complicated and inconsistent. Despite its effectiveness, on poststroke insomnia much is still unclear about the effect of Acu treatment on various sleep parameters such as subjective sleep quality, sleep latency, sleep duration etc.

Network meta-analysis can comprehensively evaluate different Acu interventions and rank their effectiveness. The objective of this study is to explore the effects of different Acu and related therapies on poststroke insomnia through network Meta-analysis and examine the degree of improvement on different sleep parameters, to provide reference for clinical practice.

## 2. Methods

Preferred Reporting Items for Systematic Reviews and Meta-Analyses for Network Meta-Analyses.^[11]^ It was registered on PROSPERO in September 2024 (registration number: CRD42024599709).

### 2.1. Search strategy

Randomized controlled trials (RCTs) of Acu and related therapies for poststroke insomnia were retrieved from 8 databases from the establishment of the database to September 2024. The databases included CNKI, Wanfang, VIP, CBM, PubMed, Embase, and Web of science and Cochrane Library. The language was limited to Chinese and English, while the literature type was limited to journals and dissertations. The search strategy used subject words combined with free words. Chinese and English search terms included “stroke,” “insomnia,” “acupuncture,” “warming acupuncture,” “electroacupuncture,” “auriculotherapy,” and “ randomized controlled trials” (see Table S1, Supplemental Digital Content 1).

### 2.2. Inclusion and exclusion criteria

#### 2.2.1. Inclusion criteria

1. Population: patients who met the diagnostic criteria for poststroke insomnia.

Insomnia should be diagnosed according to at least one set of current or past diagnostic criteria or guidelines, such as:

① Chinese Guideline for Diagnosis and Treatment of Insomnia in Adults (2017).
② Chinese Classification of Mental Disorders-3.
③ International Classification of Sleep Disorders, 3rd Edition.

1. Intervention: Acu and related therapies, including Acu, warm acupuncture (WA), electroacupuncture (Electro), auriculotherapy (AT), point embedding (PE), etc, either used alone or in combination with other therapies.
2. Comparison: Western medicine (Med), conventional rehabilitation therapy and other Acu therapies.
3. Outcomes: Pittsburgh Sleep Quality Index (PSQI) and its component dimensions, such as sleep duration, sleep latency, sleep disturbances, daytime dysfunction, subjective sleep quality and sleep efficiency.
4. Study design: RCTs.

#### 2.2.2. Exclusion criteria

1. Comparison of therapeutic effects of different Acu manipulations and acupoint selection prescriptions.
2. Clinical studies with missing data.
3. Only 1 of 2 articles with duplicate publication or data reuse were included.
4. Conference papers, case analysis, commentaries, etc.

### 2.3. Study selection and data extraction

Endnote version 21.2 was used by 2 researchers to independently screen literature and extract data according to inclusion and exclusion criteria. In case of a disagreement, a third researcher was used to break the tie. The literature was preliminarily screened by reading the title and abstract, followed by reading the full text of relevant literature. Several key data were extracted, including the name of first author, year of publication, age, gender, sample size, intervention measures, intervention period, and outcome indicators.

### 2.4. Bias risk assessment

To evaluate the risk bias, 2 investigators used RoB 2.0, which assesses the risk of bias in RCTs. Five modules from which bias may arise, include the randomization process, the intended intervention, missing outcome data, outcome measurement, and selective reporting of outcomes. The risk of bias was divided into “low risk,” “moderate risk,” and “high risk.” The 5 modules were evaluated separately, allowing for an assessment of the overall bias of the included RCTs studies.

### 2.5. Statistical analysis

Network meta-analysis was performed using R version 4.4.1 and Stata version 18.0. The mean difference was used as the effect index for the PSQI score, and its 95% confidence interval was calculated. The overall consistency was examined by testing the closed-loop consistency. When *P *> .05, there was inconsistency, however the inconsistency factor and 95% starting point were both 0, indicating better consistency. The splitting node method was used to test the regional consistency, with *P* > .05 indicated that there was no local inconsistency. If there was inconsistency, the inconsistency model was applied; otherwise, the consistency model was applied. Treatment effects were ranked using as the surface under the cumulative ranking curve, where higher values indicated better intervention effects. The closer the surface under the cumulative ranking curve value is to 100%, the better the effect of the intervention. Funnel plots were used to identify small sample effects between studies and test for publication bias. A *P*-value < 0.05 was deemed statistically significant. Finally, we used GRADE working group guidance to evaluate the certainty of evidence (see Table S2, Supplemental Digital Content 2).

## 3. Results

### 3.1. Study search and description

A total of 2094 scientific publications were retrieved from databases. Of these, 1144 and 704 were excluded for being duplicate and irrelevant literatures, respectively, leaving 246 literatures that met the threshold for initial screening. Upon rescreening, the full texts of 2 literatures were unobtainable, 95 were found to be inconsistent with this study’s objectives, while 42 had no relevant data (see Table S3, Supplemental Digital Content 3). Finally, 107 RCTs^[12–118]^ were included. The basic characteristics of the included studies are summarized in Table S4, Supplemental Digital Content 4.

The 107 RCTs, had a total of 8062 patients, of whom 54% were males and 46% females, with a mean age of 62.10 ± 9.60 years. There were 16 interventions, including 5 single Acu therapies – Acu, Electro, WA, AT, and PE – and 10 kinds of comprehensive Acu therapies, including Acu + AT, Acu + Electro, Acu + Med, Acu + PE, Acu + Electro + AT, Acu + AT + Med, AT + Med, Electro + Med, WA + AT, and WA + Med.

### 3.2. Results of bias risk assessment

**Figure 1.**
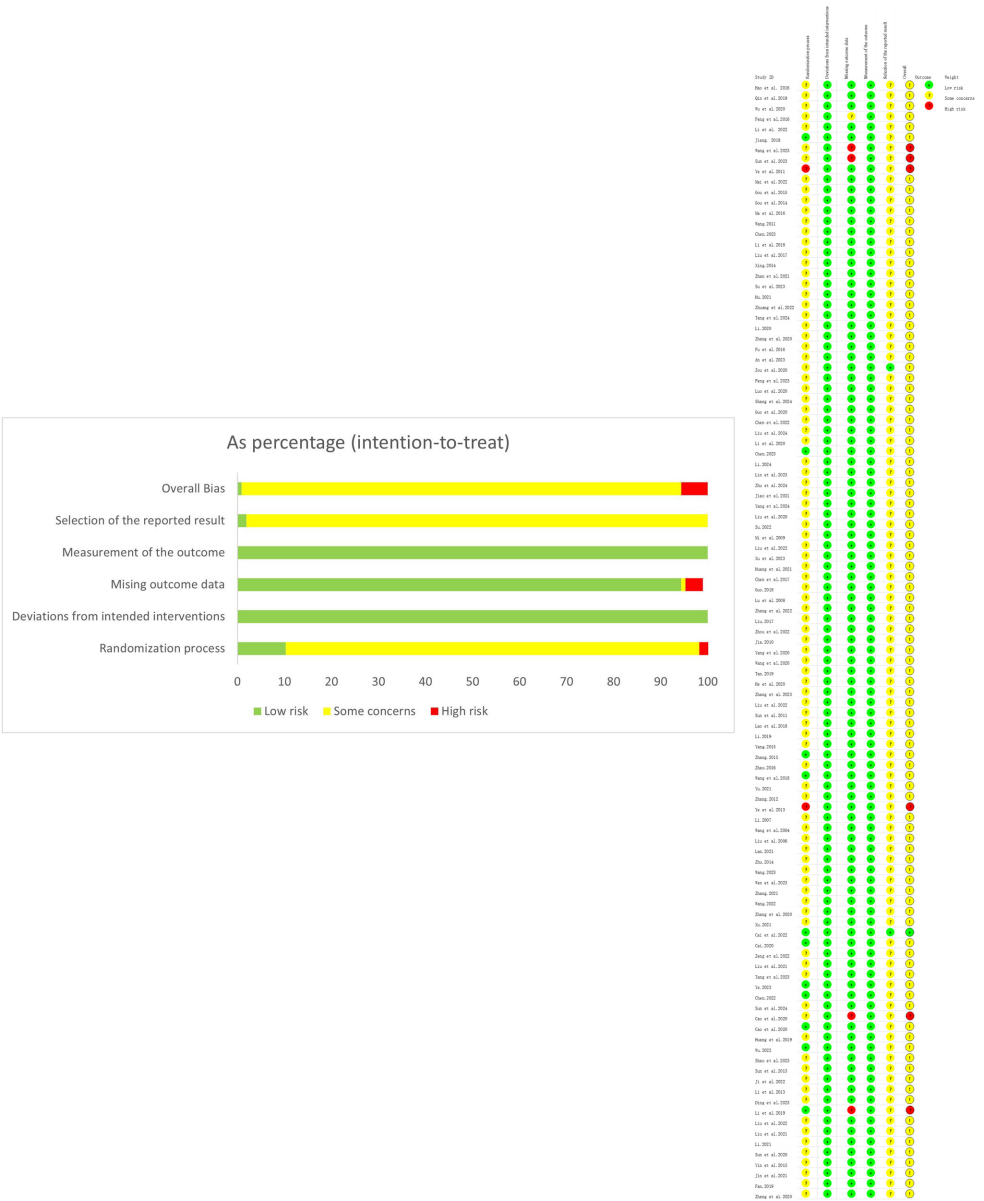
Risk of bias graph.

Of the 107 studies, 1 (0.9%) had a low risk for overall bias, 6 (5.6%) had a high risk and 100 (93.5%) were found to be having “some concerns” (Fig. 1).

### 3.3. Network meta-analysis

#### 3.3.1. PSQI total scores

The nodes in the network plots represent the different interventions, the size represents the sample size, the lines between the nodes represent the comparison of the interventions, and the width represents the number of included RCTS.

**Figure 2.**
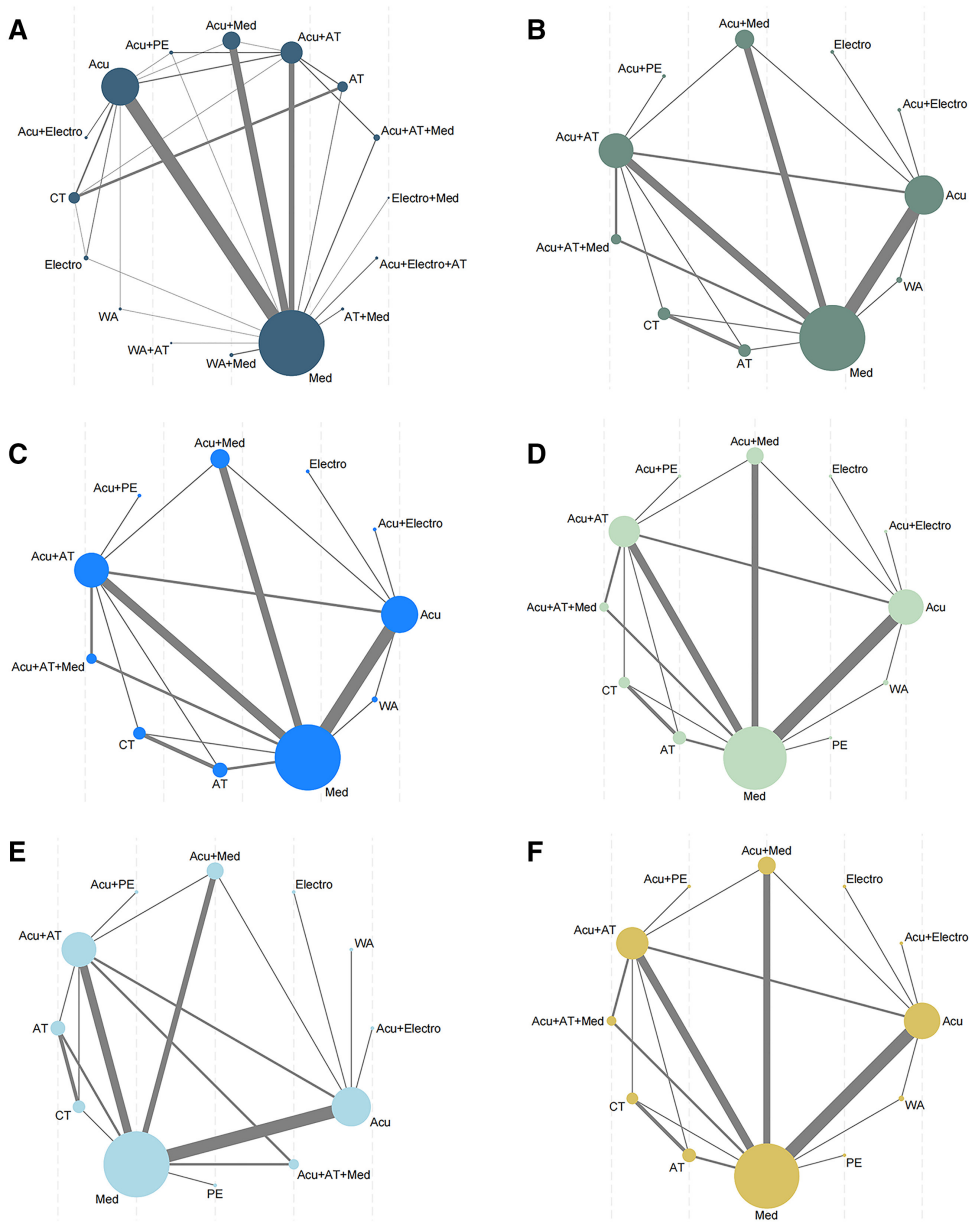
Network plot. (A) PSQI total scores; (B) sleep duration; (C) sleep latency; (D) sleep efficiency and subjective sleep quality; (E) daytime dysfunction; and (F) sleep disturbances. Acu = acupuncture, AT = auriculotherapy, CT = conventional treatment, Electro = electroacupuncture, Med = western medicine, PE = point embedding, PSQI = Pittsburgh Sleep Quality Index, WA = warm acupuncture.

**Figure 3.**
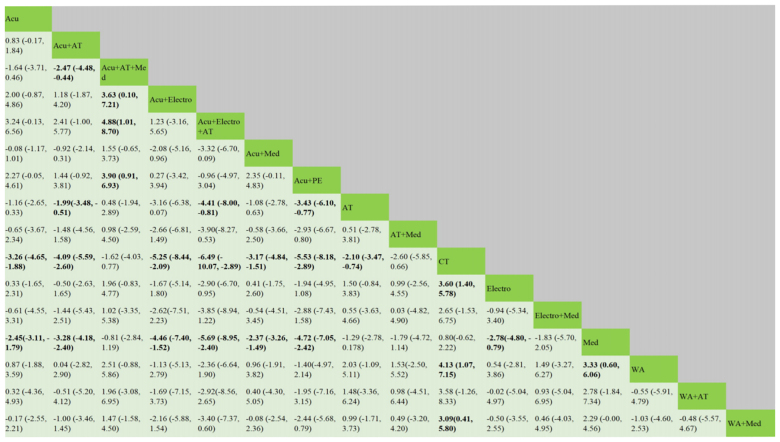
Network meta-analysis results for PSQI total scores. The bold font indicates a statistical difference. Acu = acupuncture, AT = auriculotherapy, CT = conventional treatment, Electro = electroacupuncture, Med = western medicine, PE = point embedding, PSQI = Pittsburgh Sleep Quality Index, WA = warm acupuncture.

**Figure 4.**
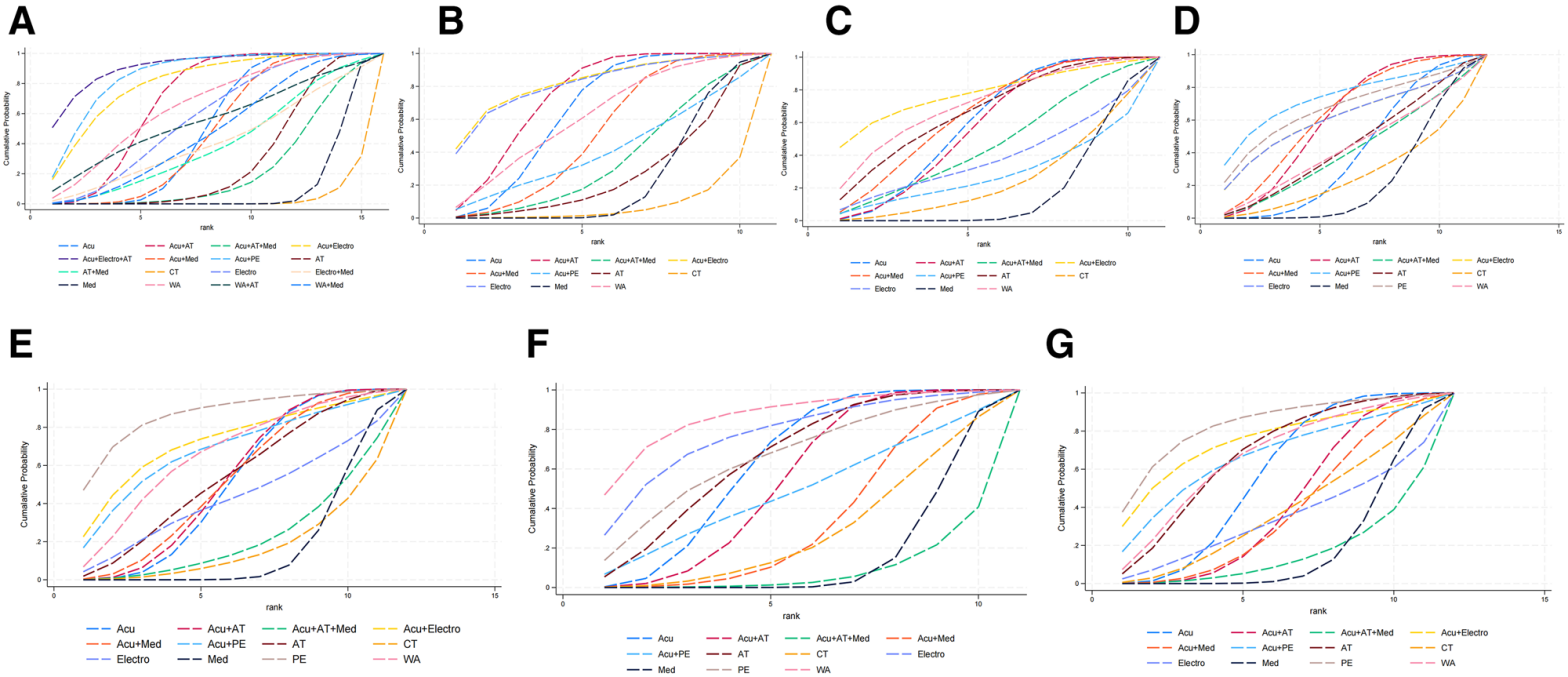
Surface under the cumulative ranking curve. (A) PSQI total scores; (B) sleep duration; (C) sleep latency; (D) sleep efficiency; (E) subjective sleep quality; (F) daytime dysfunction; and (G) sleep disturbances. Acu = acupuncture, AT = auriculotherapy, CT = conventional treatment, Electro = electroacupuncture, Med = western medicine, PE = point embedding, PSQI = Pittsburgh Sleep Quality Index, WA = warm acupuncture.

A total of 100^[12–25,27–34,36–38,41–69,71–109,112–118]^ studies reported PSQI total scores, involving 14 treatment methods, such as Acu, Electro, AT, WA, Acu + AT, Acu + Electro, Acu + Med, Acu + PE, Acu + Electro + AT, Acu + AT + Med, AT + Med, Electro + Med, WA + AT, and WA + Med. Most studies compared Acu with Med, followed by Acu + Med with Med and Acu + AT with Med. A total of 27 direct comparisons as well as 10 closed loops were formed, indicating that both direct and indirect comparison of interventions were involved (Fig. 2A). The results showed that the PSQI scores of Acu, Acu + AT, Acu + Electro, Acu + Electro + AT, Acu + Med, Acu + PE, Electro and WA were significantly lower than those of Med (*P *< .05; Fig. 3). This indicated that these treatments had better outcomes compared with Med in the treatment of poststroke insomnia. The ranking results revealed the 6 most effective interventions in reducing the PSQI total scores, they included Acu + Electro + AT (91.47%), Acu + PE (86.07%), Acu + Electro (81.2%), Acu + AT (69.49%), WA (65.88%), and Electro (57.55%, Fig. 4A).

#### 3.3.2. Sleep duration and sleep latency

**Figure 5.**
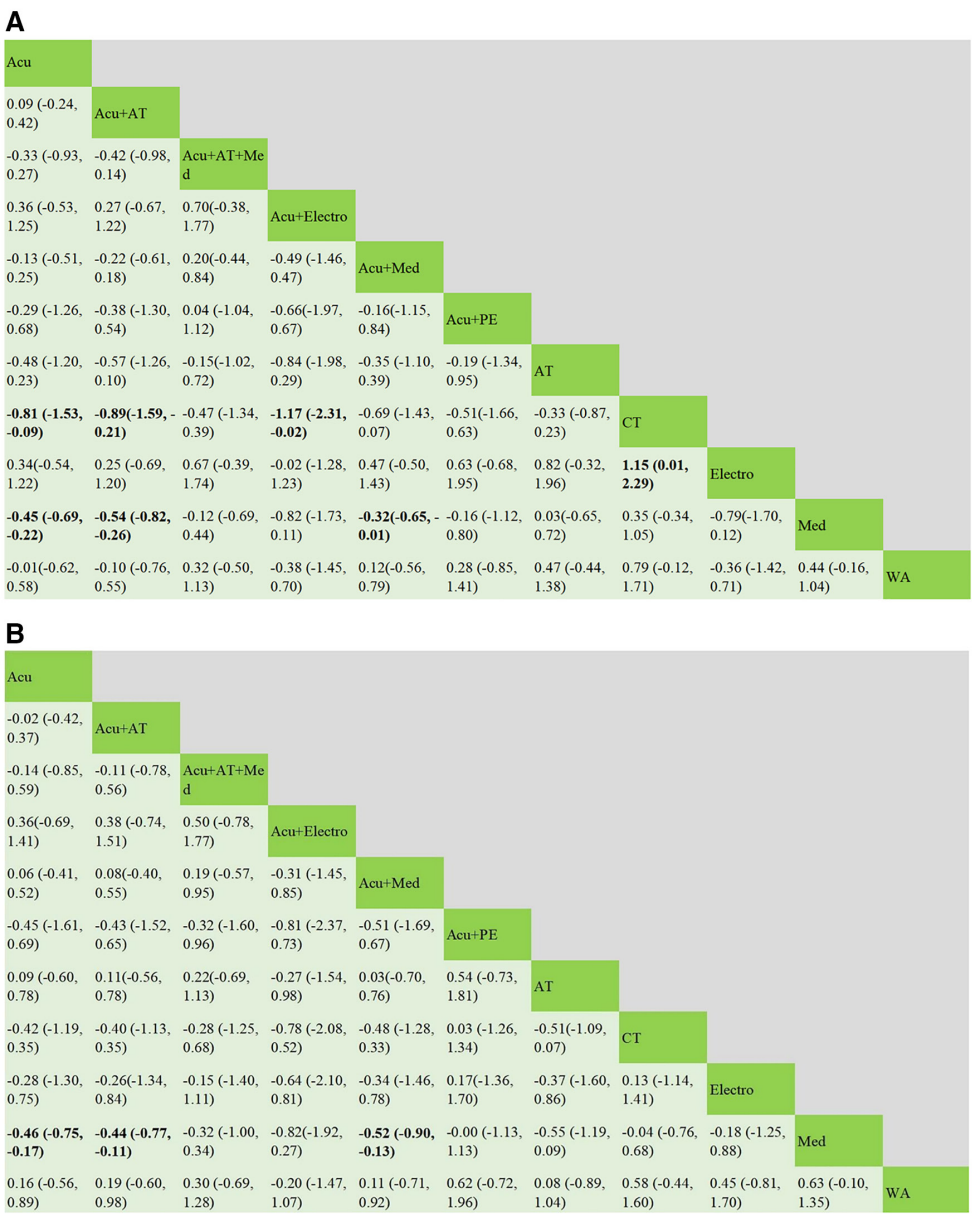
Network meta-analysis results for sleep duration and sleep latency. (A) Sleep duration and (B) sleep latency. Acu = acupuncture, AT = auriculotherapy, CT = conventional treatment, Electro = electroacupuncture, Med = western medicine, PE = point embedding, WA = warm acupuncture.

A total of 34 studies investigated sleep duration^[17,23,26,27,29,31,32,36,39–44,50,60,63,64,66,70,71,73,76–78,88,89,95,98,101,105,110–112]^ and sleep latency^[17,23,26,27,29,31,32,36,39–44,50,60,63,64,66,70,73,76–78,88,89,91,95,98,101,105,110–112]^ encompassing 9 interventions, such as Acu, AT, Electro, WA, Acu + AT, Acu + AT + Med, Acu + Electro, Acu + Med, and Acu + PE. The majority of these studies compared Acu with Med, Acu + Med with Med and Acu + AT with Med (Fig. 2B and C). Eighteen direct comparisons as well as 7 closed loops were formed. The results indicated that Acu, Acu + AT, and Acu + Med prolonged sleep duration (Fig. 5A) and shortened sleep latency (Fig. 5B) more than Med alone. Acu, Acu + AT, Acu + Electro, and Electro prolonged sleep duration more than conventional treatment (CT). The differences were statistically significant (*P* < .05). The ranking results indicated that the most effective interventions in prolonging sleep duration and shortening sleep latency, respectively, were Acu + Electro (82.38% and 77.92%), Acu + AT (74.36% and 54.37%), Acu (64.93% and 59.17%), WA (61.92% and 70.62%), and Acu + Med (52.02% and 64.89%, Fig. 4B and C). Electro and AT were also highly ranked, in prolonging sleep duration (81.47%) and shortening sleep latency (66.71%), respectively.

#### 3.3.3. Sleep efficiency and subjective sleep quality

**Figure 6.**
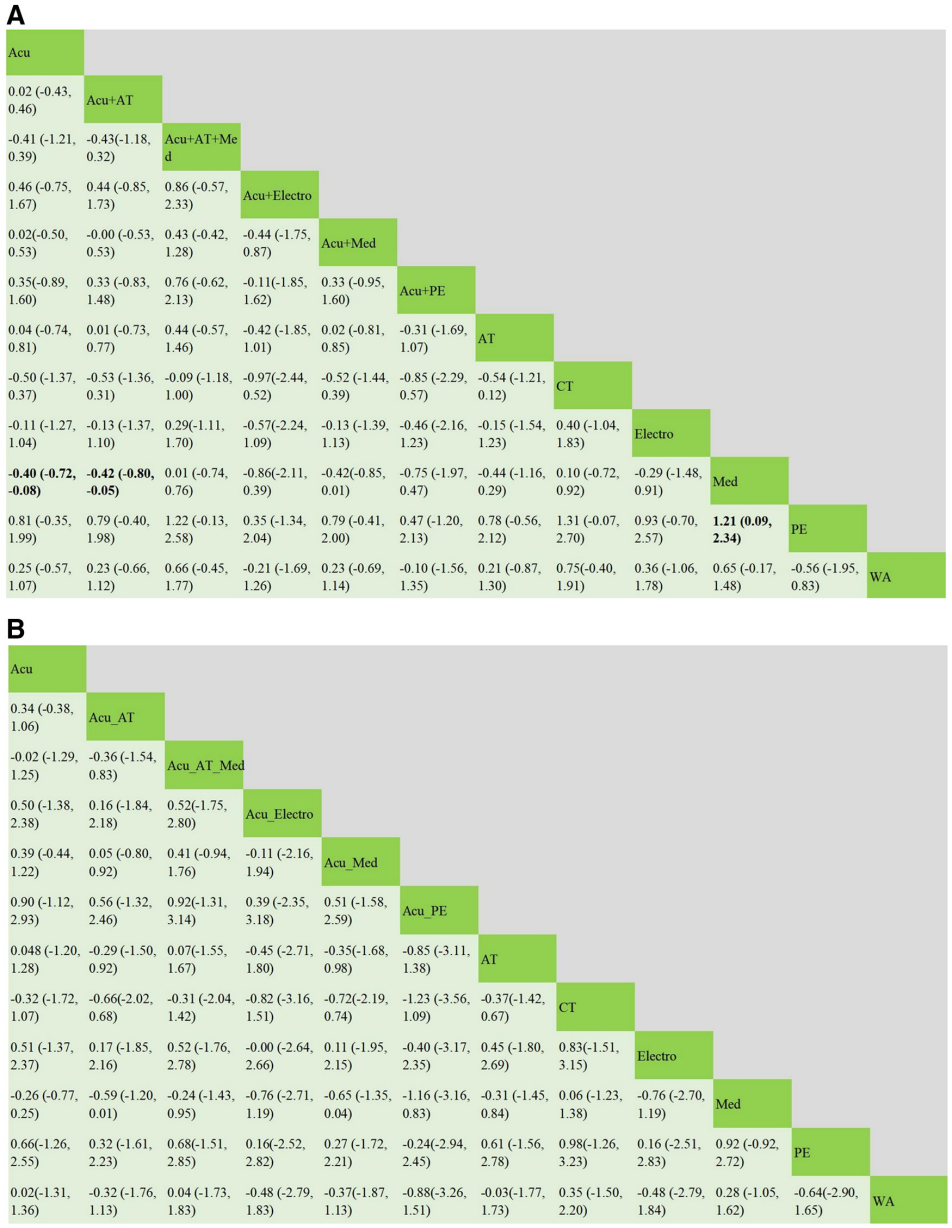
Network meta-analysis results for sleep efficiency and subjective sleep quality. (A) Subjective sleep quality and (B) sleep efficiency. Acu = acupuncture, AT = auriculotherapy, CT = conventional treatment; Electro = electroacupuncture, Med = western medicine, PE = point embedding, WA = warm acupuncture.

Sleep efficiency and subjective sleep quality were reported in 36 studies,^[17,23,26,27,29,31,32,36,39–44,50,60,63,64,66,70,71,73,76–78,88,89,91,95,98,101,105,110–112]^ involving 10 interventions, such as Acu, AT, Electro, PE, WA, Acu + AT, Acu + AT + Med, Acu + Electro, Acu + Med, and Acu + PE (Fig. 2D). Majority of the studies compared between Acu and Med, followed by Acu + Med and Med, and Acu + AT and Med. These formed 19 direct comparisons and 7 closed loops. Acu, Acu + AT and PE were better than Med in the improvement of subjective sleep quality, and the difference was statistically significant (*P *< .05, Fig. 6A). The top 6 interventions were PE (86.94%), Acu + Electro (72.39%), Acu + PE (67.63%), WA (65.82%), AT (53.43%), and Acu + AT (52.58%, Fig. 4E). The interventions did not show any significant differences in improving sleep efficacy (Fig. 6B). The 6 most effective interventions were Acu + PE (73.78%), PE (66.76%), Acu + Med (62.99%), Electro (60.89%), Acu + Electro (60.97%), and Acu + AT (60.76%, Fig. 4D).

#### 3.3.4. Daytime dysfunction

**Figure 7.**
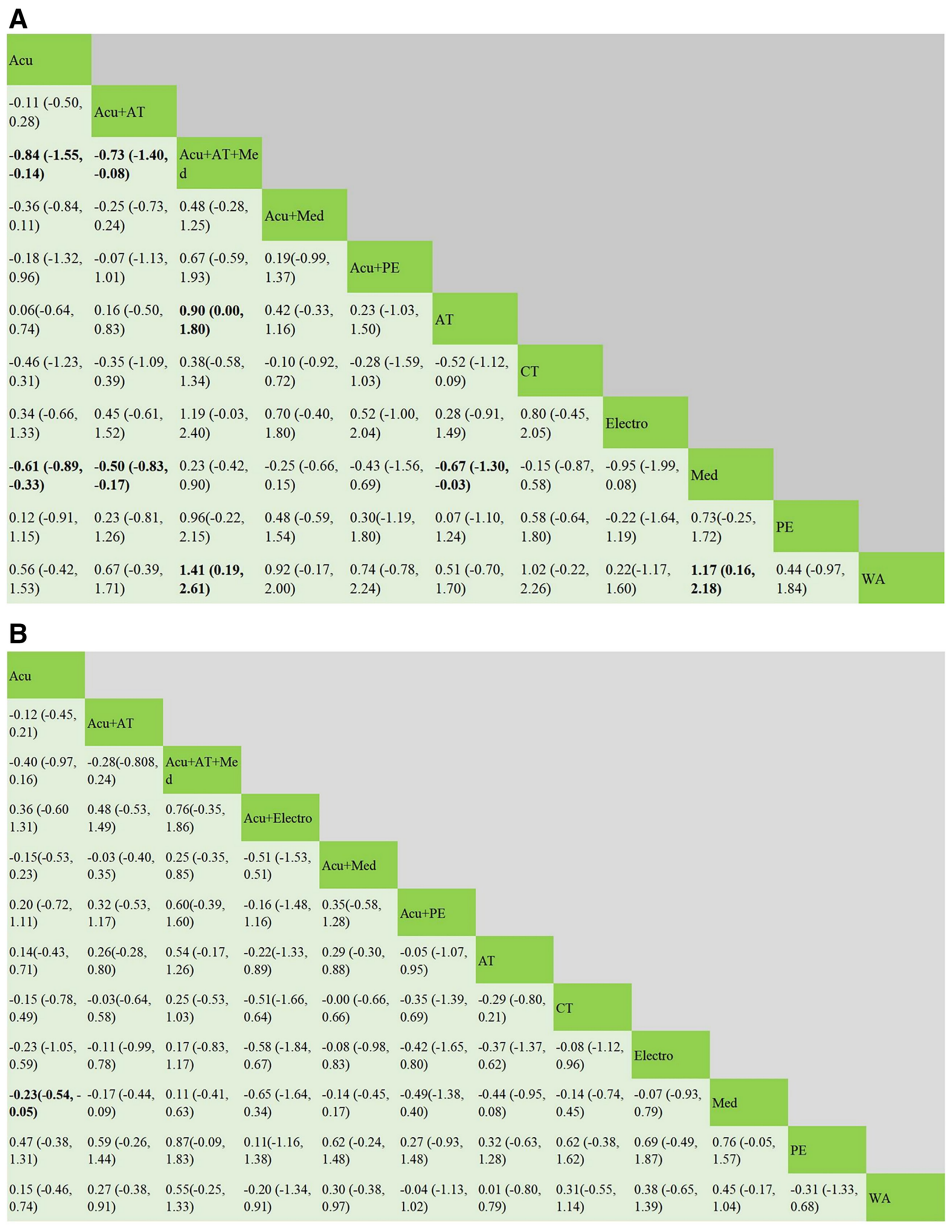
Network meta-analysis results for daytime dysfunction and sleep disturbances. (A) Daytime dysfunction and (B) sleep disturbances. Acu = acupuncture, AT = auriculotherapy, CT = conventional treatment, Electro = electroacupuncture, Med = western medicine, PE = point embedding, WA = warm acupuncture.

A total of 34 studies^[17,23,26,27,29,31,32,35,36,39–44,50,60,63,64,66,70,71,73,76–78,88,89,91,95,98,101,105,112]^ reported daytime dysfunction and involved 9 interventions, such as Acu, AT, Electro, PE, WA, Acu + AT, Acu + AT + Med, Acu + Med, and Acu + PE. Most of the studies compared Acu with Med, followed by Acu + Med with Med and Acu + AT with Med (Fig. 2E). Eighteen direct comparisons as well as 7 closed loops were formed. The results showed that Acu, Acu + AT, AT, and WA were significantly more effective than Med in improve daytime dysfunction (*P* < .05, Fig. 7A). The 6 most effective interventions in improving daytime dysfunction were WA (86.66%), Electro (77.39%), AT (66.47%), PE (66.44%), Acu (63.49%), and Acu + AT (54.37%, Fig. 4F).

#### 3.3.5. Sleep disturbances

A total of 34 studies^[17,23,26,27,29,31,32,35,36,39–44,50,60,63,66,70,73,76–78,88,89,91,95,98,101,105,110–112]^ reported sleep disturbances, involved 10 interventions, such as Acu, AT, Electro, PE, WA, Acu + AT, Acu + AT + Med, Acu + Electro, Acu + Med, and Acu + PE. Most studies compared Acu with Med, followed by Acu + Med with Med and Acu + AT with Med (Fig. 2F). Nineteen direct comparisons as well as 7 closed loops were formed. The results showed that Acu was significantly more effective in relieving sleep disturbances than Med (*P <* .05, Fig. 7B). The 6 most effective interventions in relieving sleep disturbances included PE (83.16%), Acu + Electro (74.75%), AT (67.70%), WA (66.40%), Acu + PE (66.24%), and Acu (56.25%, Fig. 4G).

### 3.4. Subgroup analysis

**Figure 8.**
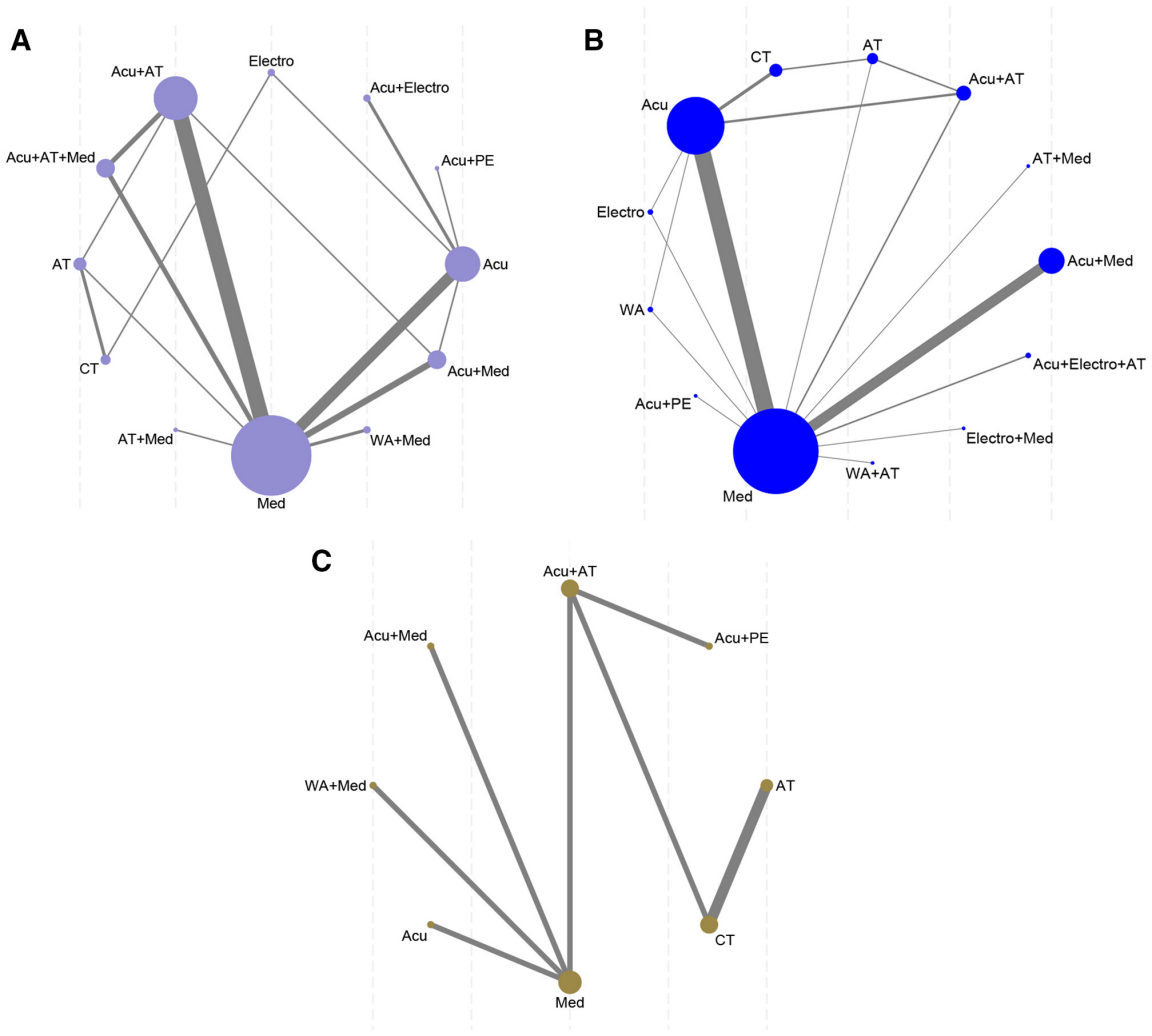
Network plot of the PSQI total score by treatment duration. (A) < 4 weeks; (B) 4 weeks; and (C) >4 weeks. Acu = acupuncture, AT = auriculotherapy, CT = conventional treatment, Electro = electroacupuncture, Med = western medicine, PE = point embedding, PSQI = Pittsburgh Sleep Quality Index, WA = warm acupuncture.

**Figure 9.**
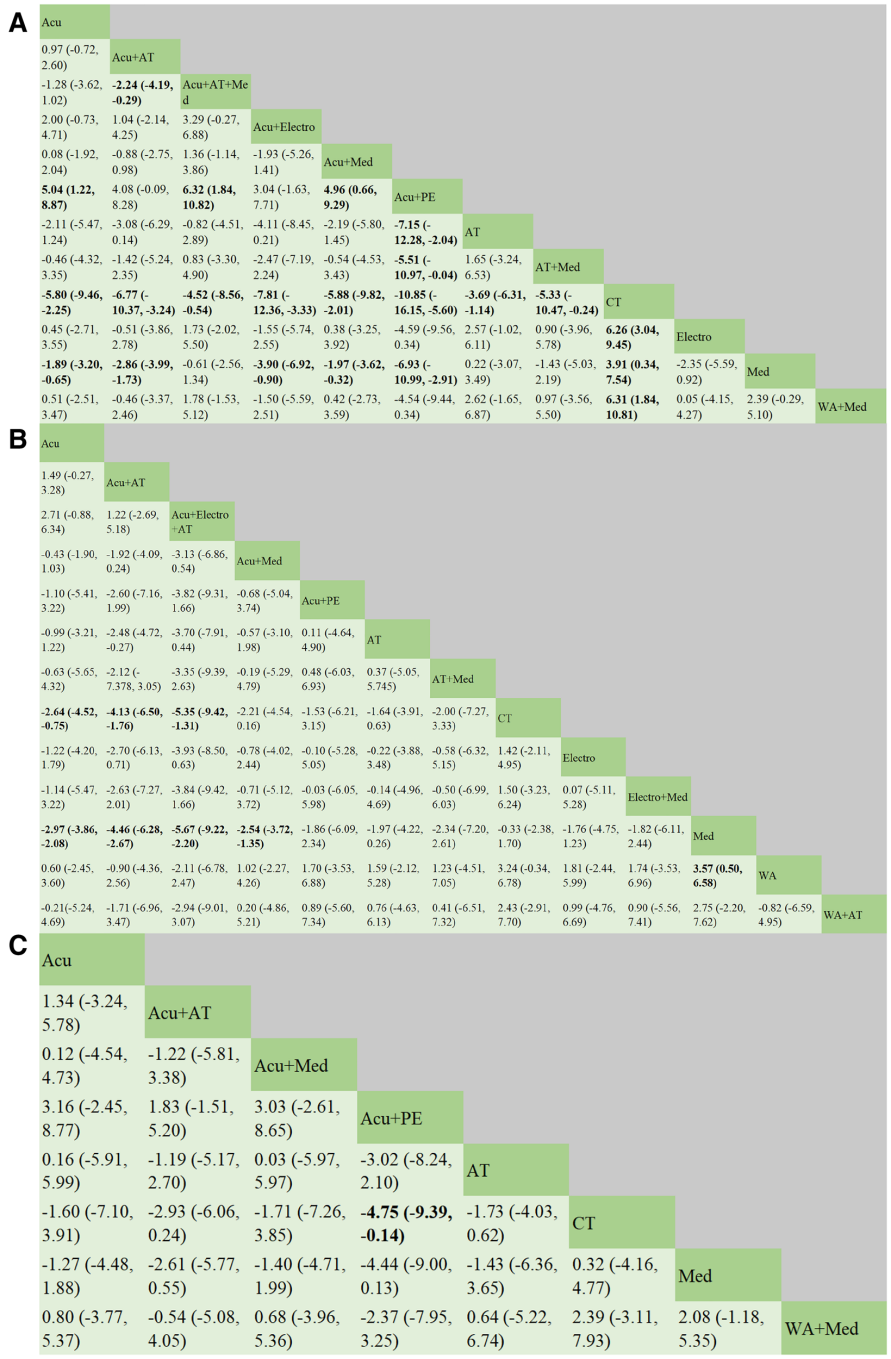
Network meta-analysis of the PSQI total score comparisons stratified by treatment duration. (A) < 4 weeks; (B) 4 weeks; and (C) >4 weeks. Acu = acupuncture, AT = auriculotherapy, CT = conventional treatment, Electro = electroacupuncture, Med = western medicine, PE = point embedding, PSQI = Pittsburgh Sleep Quality Index, WA = warm acupuncture.

**Figure 10.**
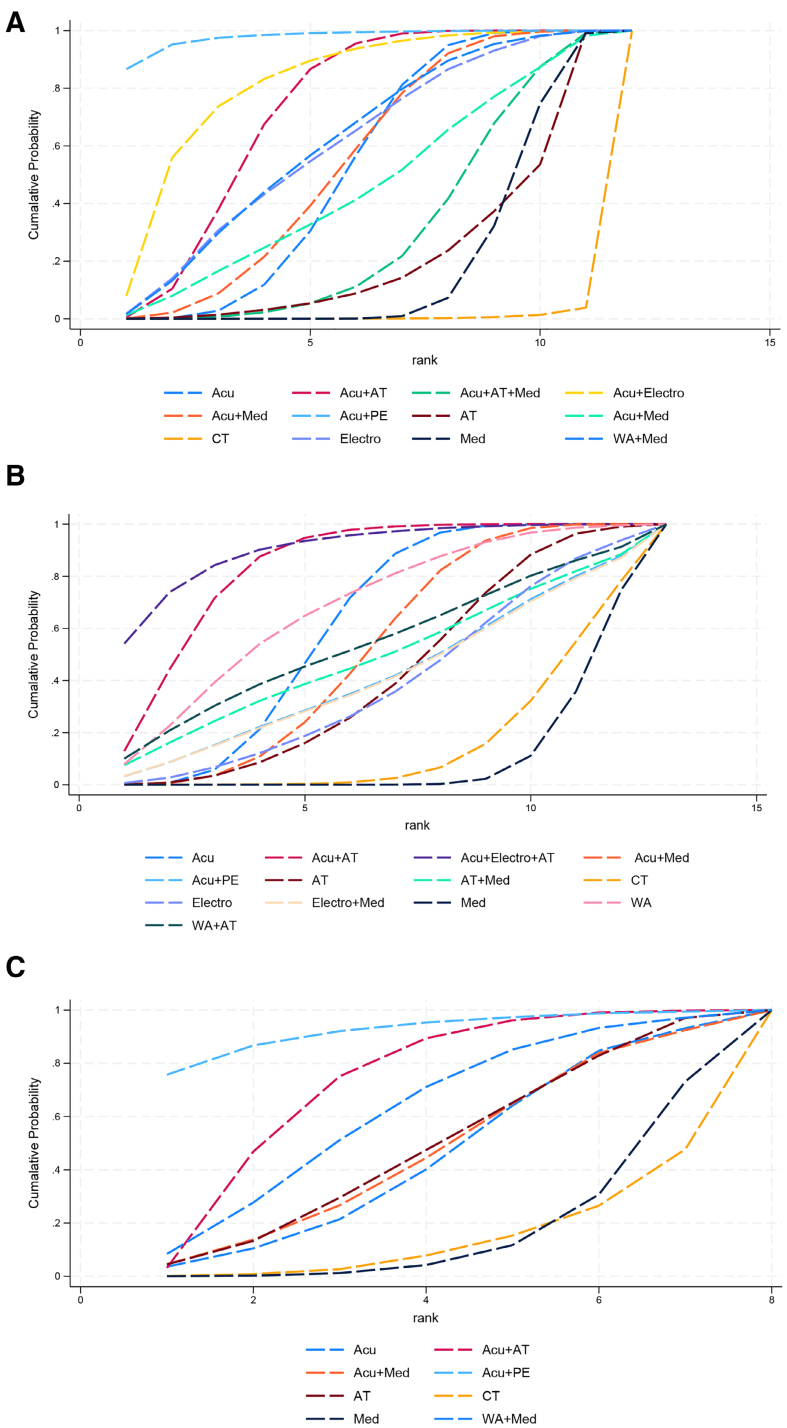
Surface under the cumulative ranking curve of the PSQI total score by treatment duration. (A) < 4 weeks; (B) 4 weeks; and (C)>4 weeks. Acu = acupuncture, AT = auriculotherapy, CT = conventional treatment, Electro = electroacupuncture, Med = western medicine, PE = point embedding, PSQI = Pittsburgh Sleep Quality Index, WA = warm acupuncture.

To explore potential sources of heterogeneity, we performed subgroup analysis of PSQI total score based on treatment duration. Among the included studies, treatment courses lasted < 4 weeks in 33,^[16,17,22,24,27–29,31,34,36,38,43–46,60,62,68,69,76,78,83,84,91,94–96,100,105,107,112,115,117]^ 4 weeks in 58,^[12,14,15,18–21,23,25,30,32,37,41,42,47–50,52–59,61,63–67,71–73,80–82,85–90,92,93,97–99,101–104,106,109,113,114,116,118]^ and > 4 weeks in 8.^[13,33,51,74,75,77,79,108]^ Studies with treatment courses < 4 weeks involved 10 interventions, forming 16 direct comparisons and 3 closed loops (Fig. 8A). Those with 4-week courses comprised 11 interventions, yielding 17 direct comparisons and 4 closed loops (Fig. 8B). Most studies compared Acu with Med. Studies with > 4-week courses included 7 interventions and 7 direct comparisons, with most comparing AT with CT (Fig. 8C). The results showed that Acu, Acu + AT, Acu + Electro, Acu + Med, Acu + PE, and WA + Med were significantly more effective than Med or CT when the treatment duration is <4 weeks (*P* < .05, Fig. 9A). The ranking results reveal the 5 most effective intervention in reducing the PSQI total scores included Acu + PE (97.81%), Acu + Electro (81.66%), Acu + AT (72.51%), WA + Med (61.50%), and Electro (60.37%; Fig. 10A), there were no significant differences in therapeutic efficacy (*P* > .05, Fig. 9A). At the 4-week mark of the treatment course, Acu, Acu + AT, and Acu + Electro + AT were more effective than Med or CT (*P*<.05, Fig. 9B). The ranking results reveal the 5 most effective included Acu + Electro + AT (90.53%), Acu + AT (83.99%), WA (68.28%), Acu (60.95%), and WA + AT (54.19%; Fig. 10B). When the treatment course exceeds 4 weeks, Acu + PE significantly reduces PSQI compared to CT (*P* < .05, Fig. 9C). The ranking results reveal the 5 most effective included Acu + PE (92.26%), Acu + AT (72.64%), WA + Med (61.67%), AT (48.69%), and Acu + Med (47.80%; Fig. 10C).

### 3.5. Reporting biases

**Figure 11.**
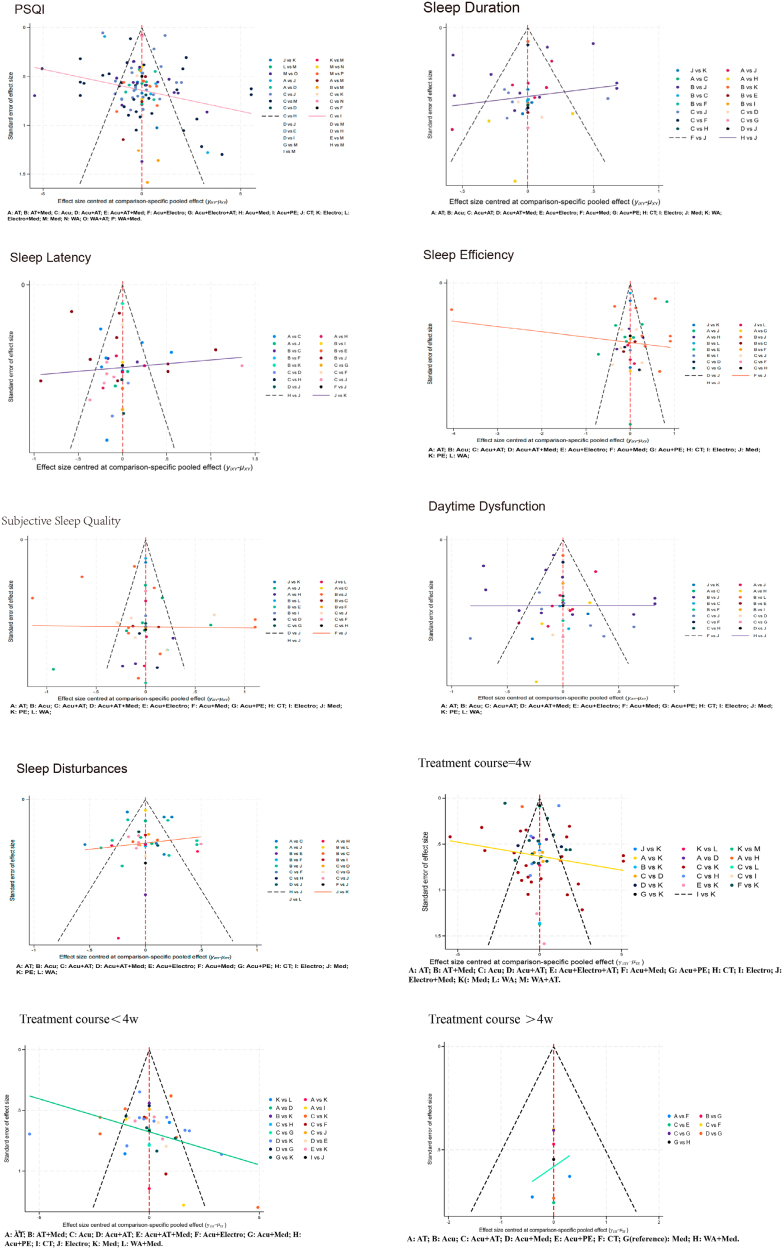
Funnel plot. Acu = acupuncture, AT = auriculotherapy, CT = conventional treatment, Electro = electroacupuncture, Med = western medicine, PE = Point embedding, PSQI = Pittsburgh Sleep Quality Index, WA = warm acupuncture.

The funnel plot showed a certain asymmetry in the PSQI total score and each parameter. Multiple studies were scattered outside the funnel plot, indicative of a small sample effect or publication bias (Fig. 11).

## 4. Discussion

This study compared the effects of Acu and related therapies on improving sleep in patients with poststroke insomnia using network meta-analysis. The results were as follows: First, Acu, Electro, WA, Acu + AT, Acu + AT + Electro, Acu + Electro, Acu + Med, Acu + PE, WA + Med were significantly better in reducing PSQI total score, than Med or CT. Combined therapies had significantly better therapeutic effect, among which Acu + AT + Electro produced best outcomes. Second, Acu, Acu + AT, and Acu + Med were more effective in prolonging sleep duration and shortening sleep latency. Third, Acu, Acu + AT and PE were more effective in improving subjective sleep quality, with PE producing the best outcomes. Fourth, Acu, Acu + AT, AT, and WA are more effective in improve daytime dysfunction, with WA having the best effect in this regard. Finally, Acu was the most effective in reducing the degree of sleep disturbances.

Studies have shown that about 40% of stroke patients meet the diagnostic criteria of poststroke insomnia, or present with insomnia related symptoms.^[119]^ There are 2 main treatments for poststroke insomnia: medication, mainly benzodiazepines, whose long-term use can result in adverse side effects; and cognitive behavioral therapy which is affected by poor patient compliance which makes it difficult to achieve the best effect. The efficacy and safety of Acu and related therapies in the treatment of poststroke insomnia are worthy of mention. Previous studies have shown that Acu and related therapies, have long-term benefits, can reduce the PSQI total score in patients with poststroke insomnia, and have better curative effects compared to oral drugs such as diazepam.^[10,120]^ When combined with other therapies, Acu has been shown to have a synergistic effect, producing even better outcomes. The mechanism Acu and related therapies in the treatment of poststroke insomnia is through the regulation of the neuro-endocrine-immune network. They regulate cerebrovascular reactivity, promote cerebral vasodilatation, increase cerebral blood flow thereby.^[22]^ Insomnia after stroke results from the imbalance of neurotransmitters. Acu and related therapies help to reduce the levels of norepinephrine, dopamine, orexin-A and serum substance P, weakening their inhibitory effect on sleep, and increase the levels of 5-hydroxytryptamine, neuropeptide Y and other neurotransmitters that promote sleep.^[48,85,90,121]^ These cumulatively result to improve the quality of sleep, effectively alleviate the clinical symptoms such as difficulty falling asleep and easy to wake up from multiple dreams. Acu can also mediate the production of melatonin, thereby regulating the circadian rhythm.^[122]^ There is an increasing amount of evidence suggesting that immune-related inflammatory factors are involved in sleep regulation.^[123]^ Electro was shown to reduce levels of interleukin-6 and tumor necrosis factor-α, which are exponentially related to severity of insomnia.^[124,125]^

Analysis of different dimensions of PSQI, revealed that distinct Acu modalities exhibited differential therapeutic effects across dimensions, primarily attributable to heterogeneity in mechanisms of action, modality-specific stimulation parameters, and targeted pathological pathways. Acu + AT played an important role in the improvement of each index. In traditional Chinese medicine, the ear is closely related to the meridians and zangfu, so the effect of dredging them can be reached by stimulating the ear acupoints. Stimulating the commonly used auricular points in the treatment of insomnia, including shenmen, subcortical, and heart, can inhibit the excitation of the cerebral cortex and reduce the secretion of neurotransmitters such as norepinephrine.^[75]^ PE utilizes absorbable threads that continuously release biological stimulation at Acu points, forming a long-lasting needling sensation that provides sustained regulation of the acupoints. This continuous stimulation repeatedly activates the hypothalamic–pituitary–adrenal axis, thereby correcting stress-induced dysregulation and thus more significantly alleviating patients’ subjective sleep quality.^[126]^ WA is an intervention that combines Acu and moxibustion, which has the effect of warming meridians and promoting the circulation of qi and blood. Therefore, it enhances cellular energy metabolism, directly alleviating physical fatigue-a common manifestation of daytime dysfunction.^[127]^ However, one study showed that WA had no obvious advantage in improving PSQI total score and other dimensions compared with estazolam, with the exception of shortening the sleep latency, which may be caused by different acupoint selection.^[110]^ Although Acu and related therapies have demonstrated good effects on shortening sleep latency, prolonging sleep duration, ameliorating sleep disorders and daytime function, none in when alone or combined has shown better therapeutic effects than oral Med or conventional rehabilitation therapy in terms of sleep efficiency. This may be caused by the sleep-efficiency scoring rule that reduces the score by one point and requires patients to minimize sleep onset latency and increase sleep duration as much as possible.

After subgroup analysis of the treatment course, the results indicate that the advantages of Acu and related therapies are more pronounced when the treatment duration is less than 4 weeks, often outperforming western medication or conventional therapies. This is due to Acu and related therapies’ ability to rapidly regulate neurotransmitters, endocrine functions, and autonomic nervous system activity, effectively breaking the vicious cycle of insomnia.^[128]^ After exceeding 4 weeks, the additional advantage of Acu over western medication often diminishes, with their efficacy tending to converge. This may occur because the rapid physiological regulatory effects of Acu reach a plateau, while the body develops adaptation, yet the underlying causes of chronic insomnia remain unresolved. Consequently, Acu struggles to maintain long-term superiority, whereas western medication can sustain therapeutic effects through continuous receptor-level actions. This outcome does not negate Acu’s value but rather indirectly highlights its strengths in rapid and safe onset of action. It also offers important implications, prompting us to develop long-term management and optimization strategies, such as adjusting Acu protocols or implementing intermittent treatment, to achieve enhanced therapeutic outcomes.

Compared with previously published studies, this database search was more comprehensive and included a larger number of articles, covered almost all Acu related therapies, including combinations of different measures. A comprehensive analysis was conducted to verify the therapeutic effects of Acu and related therapies on PSQI total score and different dimension scores of poststroke insomnia. It should be noted that this study was not without limitations: First, although more than 100 articles were included, only one was in English, and the quality of the articles varied. Furthermore, based on the network’s topological structure, we observed that some connections between certain measures were very thin or located at the periphery of the network. This suggests the possibility of small sample benefits and indicates a certain publication bias in the included studies. Second, almost all the participants in the studies were Chinese, which limits the scientific validity of the results. Third, this study did not further the long-term benefits of Acu for insomnia after stroke patients. Finally, due to the different selection of Acu points, the potential for heterogeneity among the included studies was high, impacting the level of evidence.

## 5. Conclusion

Network meta-analysis indicated that Acu and related therapies can effectively improve poststroke insomnia. Additionally, the combined treatment appears to have had a synergistic effect, as Acu + AT + Electro demonstrated the best outcomes. However, in clinical application, different treatment measures should be applied on patients with different manifestations of insomnia. For example, in patients with daytime dysfunction as the main manifestation, WA can be selected, while those with poor subjective sleep quality, acupoint catgut-embedding can be added. For those with long sleep latency and short sleep duration, auricular point therapy can be added. Further studies, should focus on the standardizing and improving rigour of clinical trial protocols. This will provide more reliable evidence for acupuncture-moxibustion and related therapies to treat patients with insomnia after stroke.

## Author contributions

**Conceptualization:** Yao Xie, Junlin Jiang.

**Data curation:** Jie Tang.

**Formal analysis:** Huizhong Tan, Qian Liu.

**Funding acquisition:** Le Xie, Dahua Wu.

**Investigation:** Liangyi Xiao, Shanshan Zeng, Shiliang Wang.

**Methodology:** Junlin Jiang.

**Project administration:** Le Xie, Dahua Wu.

**Writing – original draft:** Jie Tang.

**Writing – review & editing:** Jie Tang.








