# Supplementary material for: Effectiveness of acupuncture and related therapies for post-stroke insomnia: A systematic review and Bayesian network analysis
Source: Medicine (Baltimore). 2026 Aug 7;105(32):e50081. doi: 10.1097/MD.0000000000050081 (PMC13456779; doi:10.1097/MD.0000000000050081)
Supplement: Supplementary file 1 [file medi-105-e50081-s001.docx]

**Table S1. Search strategy**

**PubMed**

| Search number | Query | Search Details | Results | Time |
| --- | --- | --- | --- | --- |
| 11 | #3 AND #6 AND #9 AND #10 | ("Stroke"[MeSH Terms] OR ("brain attack"[Title/Abstract] OR "brain insult*"[Title/Abstract] OR "brain vascular accident*"[Title/Abstract] OR "cerebral hemorrhage"[Title/Abstract] OR "cerebral infarction"[Title/Abstract] OR "cerebral insult"[Title/Abstract] OR "cerebral vascular accident"[Title/Abstract] OR "cerebral vascular insufficiency"[Title/Abstract] OR "cerebro vascular accident"[Title/Abstract] OR "cerebrovascular accident*"[Title/Abstract] OR "cerebrovascular failure"[Title/Abstract] OR "cerebrovascular injury"[Title/Abstract] OR "cerebrovascular insufficiency"[Title/Abstract] OR "cerebrovascular insult"[Title/Abstract] OR "cva"[Title/Abstract] OR "Hemorrhagic cerebral infarction"[Title/Abstract] OR "insultus cerebralis"[Title/Abstract] OR "Ischemic cerebral infarction"[Title/Abstract] OR "ischemic seizure"[Title/Abstract] OR "stroke*"[Title/Abstract])) AND ("Sleep Initiation and Maintenance Disorders"[MeSH Terms] OR "Wakefulness"[MeSH Terms] OR ("agrypnia"[Title/Abstract] OR "awake*"[Title/Abstract] OR "DIMS"[Title/Abstract] OR "Disorders of Initiating and Maintaining Sleep"[Title/Abstract] OR "Early Awakening"[Title/Abstract] OR "hyposomnia*"[Title/Abstract] OR "insomnia*"[Title/Abstract] OR "Sleep disorders"[Title/Abstract] OR "sleep initiation and maintenance disorder*"[Title/Abstract] OR "sleep initiation dysfunction*"[Title/Abstract] OR "sleeplessness"[Title/Abstract] OR "wake up threshold"[Title/Abstract] OR "wakefullness"[Title/Abstract] OR "wakefulness*"[Title/Abstract] OR "waking"[Title/Abstract] OR "insomnia"[Title/Abstract] OR "Wakefulness"[Title/Abstract])) AND ("Acupuncture"[MeSH Terms] OR "Electroacupuncture"[MeSH Terms] OR "Auriculotherapy"[MeSH Terms] OR ("ACET"[Title/Abstract] OR "acupoint catgut embedding therapy"[Title/Abstract] OR "acupuncture*"[Title/Abstract] OR "auricular needling"[Title/Abstract] OR "auriculoacupuncture"[Title/Abstract] OR "auriculotherap*"[Title/Abstract] OR "burnt needle therapy"[Title/Abstract] OR "catgut embedding"[Title/Abstract] OR "catgut implantation"[Title/Abstract] OR "ear point"[Title/Abstract] OR "Electroacupuncture"[Title/Abstract] OR "fire needle therapy"[Title/Abstract] OR "fire needling"[Title/Abstract] OR "needle knife"[Title/Abstract] OR "needle punching"[Title/Abstract] OR "Pharmacopuncture"[Title/Abstract] OR "point embedding"[Title/Abstract] OR "thermoacupuncture"[Title/Abstract] OR "warm needl*"[Title/Abstract] OR "warming"[Title/Abstract])) AND ("random*"[Title/Abstract] OR "RCT"[Title/Abstract] OR "randomized controlled trial"[Title/Abstract] OR "control*"[Title/Abstract] OR "placebo"[Title/Abstract]) | 44 | 0:23:51 |
| 10 | "random*"[Title/Abstract] OR "RCT"[Title/Abstract] OR "randomized controlled trial"[Title/Abstract] OR "control*"[Title/Abstract] OR "placebo"[Title/Abstract] | "random*"[Title/Abstract] OR "RCT"[Title/Abstract] OR "randomized controlled trial"[Title/Abstract] OR "control*"[Title/Abstract] OR "placebo"[Title/Abstract] | 5,803,714 | 0:23:27 |
| 9 | #7 OR #8 | "Acupuncture"[MeSH Terms] OR "Electroacupuncture"[MeSH Terms] OR "Auriculotherapy"[MeSH Terms] OR "ACET"[Title/Abstract] OR "acupoint catgut embedding therapy"[Title/Abstract] OR "acupuncture*"[Title/Abstract] OR "auricular needling"[Title/Abstract] OR "auriculoacupuncture"[Title/Abstract] OR "auriculotherap*"[Title/Abstract] OR "burnt needle therapy"[Title/Abstract] OR "catgut embedding"[Title/Abstract] OR "catgut implantation"[Title/Abstract] OR "ear point"[Title/Abstract] OR "Electroacupuncture"[Title/Abstract] OR "fire needle therapy"[Title/Abstract] OR "fire needling"[Title/Abstract] OR "needle knife"[Title/Abstract] OR "needle punching"[Title/Abstract] OR "Pharmacopuncture"[Title/Abstract] OR "point embedding"[Title/Abstract] OR "thermoacupuncture"[Title/Abstract] OR "warm needl*"[Title/Abstract] OR "warming"[Title/Abstract] | 73,827 | 0:23:08 |
| 8 | "ACET"[Title/Abstract] OR "acupoint catgut embedding therapy"[Title/Abstract] OR "acupuncture*"[Title/Abstract] OR "auricular needling"[Title/Abstract] OR "auriculoacupuncture"[Title/Abstract] OR "Auriculotherap*"[Title/Abstract] OR "burnt needle therapy"[Title/Abstract] OR "catgut embedding"[Title/Abstract] OR "catgut implantation"[Title/Abstract] OR "ear point "[Title/Abstract] OR "Electroacupuncture"[Title/Abstract] OR "fire needle therapy"[Title/Abstract] OR "fire needling"[Title/Abstract] OR "imbedding needle"[Title/Abstract] OR "needle knife"[Title/Abstract] OR "needle punching "[Title/Abstract] OR "Pharmacopuncture"[Title/Abstract] OR "point embedding"[Title/Abstract] OR "shonishin"[Title/Abstract] OR "thermoacupuncture"[Title/Abstract] OR "warm needl*"[Title/Abstract] OR "warming"[Title/Abstract] | "ACET"[Title/Abstract] OR "acupoint catgut embedding therapy"[Title/Abstract] OR "acupuncture*"[Title/Abstract] OR "auricular needling"[Title/Abstract] OR "auriculoacupuncture"[Title/Abstract] OR "auriculotherap*"[Title/Abstract] OR "burnt needle therapy"[Title/Abstract] OR "catgut embedding"[Title/Abstract] OR "catgut implantation"[Title/Abstract] OR "ear point"[Title/Abstract] OR "Electroacupuncture"[Title/Abstract] OR "fire needle therapy"[Title/Abstract] OR "fire needling"[Title/Abstract] OR "needle knife"[Title/Abstract] OR "needle punching"[Title/Abstract] OR "Pharmacopuncture"[Title/Abstract] OR "point embedding"[Title/Abstract] OR "thermoacupuncture"[Title/Abstract] OR "warm needl*"[Title/Abstract] OR "warming"[Title/Abstract] | 73,243 | 0:22:55 |
| 7 | (("Acupuncture"[Mesh]) OR "Electroacupuncture"[Mesh]) OR "Auriculotherapy"[Mesh] | "Acupuncture"[MeSH Terms] OR "Electroacupuncture"[MeSH Terms] OR "Auriculotherapy"[MeSH Terms] | 7,993 | 0:22:35 |
| 6 | #4 OR #5 | "Sleep Initiation and Maintenance Disorders"[MeSH Terms] OR "Wakefulness"[MeSH Terms] OR "agrypnia"[Title/Abstract] OR "awake*"[Title/Abstract] OR "DIMS"[Title/Abstract] OR "Disorders of Initiating and Maintaining Sleep"[Title/Abstract] OR "Early Awakening"[Title/Abstract] OR "hyposomnia*"[Title/Abstract] OR "insomnia*"[Title/Abstract] OR "Sleep disorders"[Title/Abstract] OR "sleep initiation and maintenance disorder*"[Title/Abstract] OR "sleep initiation dysfunction*"[Title/Abstract] OR "sleeplessness"[Title/Abstract] OR "wake up threshold"[Title/Abstract] OR "wakefullness"[Title/Abstract] OR "wakefulness*"[Title/Abstract] OR "waking"[Title/Abstract] OR "insomnia"[Title/Abstract] OR "Wakefulness"[Title/Abstract] | 122,164 | 0:19:49 |
| 5 | "agrypnia"[Title/Abstract] OR "awake*"[Title/Abstract] OR "DIMS "[Title/Abstract] OR "disorder* of sleep initiation and maintenance"[Title/Abstract] OR "Disorders of Initiating and Maintaining Sleep"[Title/Abstract] OR "Early Awakening"[Title/Abstract] OR "hyposomnia*"[Title/Abstract] OR "insomnia*"[Title/Abstract] OR "Sleep disorders"[Title/Abstract] OR "sleep initiation and maintenance disorder*"[Title/Abstract] OR "Sleep Initiation Dysfunction*"[Title/Abstract] OR "sleeplessness"[Title/Abstract] OR "wake up threshold"[Title/Abstract] OR "wakefullness"[Title/Abstract] OR "wakefulness*"[Title/Abstract] OR "waking"[Title/Abstract] OR "insomnia"[Title/Abstract] OR "wakefulness"[Title/Abstract] | "agrypnia"[Title/Abstract] OR "awake*"[Title/Abstract] OR "DIMS"[Title/Abstract] OR "Disorders of Initiating and Maintaining Sleep"[Title/Abstract] OR "Early Awakening"[Title/Abstract] OR "hyposomnia*"[Title/Abstract] OR "insomnia*"[Title/Abstract] OR "Sleep disorders"[Title/Abstract] OR "sleep initiation and maintenance disorder*"[Title/Abstract] OR "sleep initiation dysfunction*"[Title/Abstract] OR "sleeplessness"[Title/Abstract] OR "wake up threshold"[Title/Abstract] OR "wakefullness"[Title/Abstract] OR "wakefulness*"[Title/Abstract] OR "waking"[Title/Abstract] OR "insomnia"[Title/Abstract] OR "wakefulness"[Title/Abstract] | 111,228 | 0:18:58 |
| 4 | ("Sleep Initiation and Maintenance Disorders"[Mesh]) OR "Wakefulness"[Mesh] | "Sleep Initiation and Maintenance Disorders"[MeSH Terms] OR "Wakefulness"[MeSH Terms] | 39,718 | 22:43:49 |
| 3 | #1 OR #2 | "Stroke"[MeSH Terms] OR "brain attack"[Title/Abstract] OR "brain insult*"[Title/Abstract] OR "brain vascular accident*"[Title/Abstract] OR "cerebral hemorrhage"[Title/Abstract] OR "cerebral infarction"[Title/Abstract] OR "cerebral insult"[Title/Abstract] OR "cerebral vascular accident"[Title/Abstract] OR "cerebral vascular insufficiency"[Title/Abstract] OR "cerebro vascular accident"[Title/Abstract] OR "cerebrovascular accident*"[Title/Abstract] OR "cerebrovascular failure"[Title/Abstract] OR "cerebrovascular injury"[Title/Abstract] OR "cerebrovascular insufficiency"[Title/Abstract] OR "cerebrovascular insult"[Title/Abstract] OR "cva"[Title/Abstract] OR "Hemorrhagic cerebral infarction"[Title/Abstract] OR "insultus cerebralis"[Title/Abstract] OR "Ischemic cerebral infarction"[Title/Abstract] OR "ischemic seizure"[Title/Abstract] OR "stroke*"[Title/Abstract] | 408,392 | 22:43:09 |
| 2 | "brain accident"[Title/Abstract] OR "brain attack"[Title/Abstract] OR "brain blood flow disturbance"[Title/Abstract] OR "brain insult*"[Title/Abstract] OR "brain vascular accident*"[Title/Abstract] OR "cerebral hemorrhage"[Title/Abstract] OR "cerebral infarction"[Title/Abstract] OR "cerebral insult"[Title/Abstract] OR "cerebral vascular accident"[Title/Abstract] OR "cerebral vascular insufficiency"[Title/Abstract] OR "cerebro vascular accident"[Title/Abstract] OR "cerebrovascular accident*"[Title/Abstract] OR "cerebrovascular arrest"[Title/Abstract] OR "cerebrovascular failure"[Title/Abstract] OR "cerebrovascular injury"[Title/Abstract] OR "cerebrovascular insufficiency"[Title/Abstract] OR "cerebrovascular insult"[Title/Abstract] OR "cerebrum vascular accident"[Title/Abstract] OR "CVA*"[Title/Abstract] OR "Hemorrhagic cerebral infarction"[Title/Abstract] OR "insultus cerebralis"[Title/Abstract] OR "ischaemic seizure"[Title/Abstract] OR "Ischemic cerebral infarction"[Title/Abstract] OR "ischemic seizure"[Title/Abstract] OR "stroke*"[Title/Abstract] | "brain attack"[Title/Abstract] OR "brain insult*"[Title/Abstract] OR "brain vascular accident*"[Title/Abstract] OR "cerebral hemorrhage"[Title/Abstract] OR "cerebral infarction"[Title/Abstract] OR "cerebral insult"[Title/Abstract] OR "cerebral vascular accident"[Title/Abstract] OR "cerebral vascular insufficiency"[Title/Abstract] OR "cerebro vascular accident"[Title/Abstract] OR "cerebrovascular accident*"[Title/Abstract] OR "cerebrovascular failure"[Title/Abstract] OR "cerebrovascular injury"[Title/Abstract] OR "cerebrovascular insufficiency"[Title/Abstract] OR "cerebrovascular insult"[Title/Abstract] OR "cva"[Title/Abstract] OR "Hemorrhagic cerebral infarction"[Title/Abstract] OR "insultus cerebralis"[Title/Abstract] OR "Ischemic cerebral infarction"[Title/Abstract] OR "ischemic seizure"[Title/Abstract] OR "stroke*"[Title/Abstract] | 374,804 | 22:42:49 |
| 1 | "Stroke"[Mesh] | "Stroke"[MeSH Terms] | 185,024 | 22:42:26 |

**Embase**

| No. | Query | Results | Date |
| --- | --- | --- | --- |
| #11 | #3 AND #6 AND #9 AND #10 | 94 | 2024/9/30 |
| #10 | 'random*':ti,kw,ab OR 'rct':ti,kw,ab OR 'randomized controlled trial':ti,kw,ab OR 'control*':ti,kw,ab OR 'placebo':ti,kw,ab | 7628499 | 2024/9/30 |
| #9 | #7 OR #8 | 108020 | 2024/9/30 |
| #8 | 'acet':ti,kw,ab OR 'acupoint catgut embedding therapy':ti,kw,ab OR 'acupuncture*':ti,kw,ab OR 'auricular needling':ti,kw,ab OR 'auriculoacupuncture':ti,kw,ab OR 'auriculotherap*':ti,kw,ab OR 'burnt needle therapy':ti,kw,ab OR 'catgut embedding':ti,kw,ab OR 'catgut implantation':ti,kw,ab OR 'ear point':ti,kw,ab OR 'electroacupuncture':ti,kw,ab OR 'fire needle therapy':ti,kw,ab OR 'fire needling':ti,kw,ab OR 'imbedding needle':ti,kw,ab OR 'needle knife':ti,kw,ab OR 'needle punching':ti,kw,ab OR 'pharmacopuncture':ti,kw,ab OR 'point embedding':ti,kw,ab OR 'shonishin':ti,kw,ab OR 'thermoacupuncture':ti,kw,ab OR 'warm needl*':ti,kw,ab OR 'warming':ti,kw,ab | 90250 | 2024/9/30 |
| #7 | 'acupuncture'/exp OR 'auricular acupuncture'/exp OR 'catgut embedding'/exp | 61065 | 2024/9/30 |
| #6 | #4 OR #5 | 224626 | 2024/9/30 |
| #5 | 'agrypnia':ti,kw,ab OR 'awake*':ti,kw,ab OR 'dims':ti,kw,ab OR 'disorder* of sleep initiation and maintenance':ti,kw,ab OR 'disorders of initiating and maintaining sleep':ti,kw,ab OR 'early awakening':ti,kw,ab OR 'hyposomnia*':ti,kw,ab OR 'insomnia*':ti,kw,ab OR 'sleep disorders':ti,kw,ab OR 'sleep initiation and maintenance disorder*':ti,kw,ab OR 'sleep initiation dysfunction*':ti,kw,ab OR 'sleeplessness':ti,kw,ab OR 'wake up threshold':ti,kw,ab OR 'wakefullness':ti,kw,ab OR 'wakefulness*':ti,kw,ab OR 'waking':ti,kw,ab OR 'insomnia':ti,kw,ab OR 'wakefulness':ti,kw,ab | 170645 | 2024/9/30 |
| #4 | 'wakefulness'/exp OR 'insomnia'/exp | 137647 | 2024/9/30 |
| #3 | #1 OR #2 | 711559 | 2024/9/30 |
| #2 | 'brain accident':ti,kw,ab OR 'brain attack':ti,kw,ab OR 'brain blood flow disturbance':ti,kw,ab OR 'brain insult*':ti,kw,ab OR 'brain vascular accident*':ti,kw,ab OR 'cerebral hemorrhage':ti,kw,ab OR 'cerebral infarction':ti,kw,ab OR 'cerebral insult':ti,kw,ab OR 'cerebral vascular accident':ti,kw,ab OR 'cerebral vascular insufficiency':ti,kw,ab OR 'cerebro vascular accident':ti,kw,ab OR 'cerebrovascular accident*':ti,kw,ab OR 'cerebrovascular arrest':ti,kw,ab OR 'cerebrovascular failure':ti,kw,ab OR 'cerebrovascular injury':ti,kw,ab OR 'cerebrovascular insufficiency':ti,kw,ab OR 'cerebrovascular insult':ti,kw,ab OR 'cerebrum vascular accident':ti,kw,ab OR 'cva*':ti,kw,ab OR 'hemorrhagic cerebral infarction':ti,kw,ab OR 'insultus cerebralis':ti,kw,ab OR 'ischaemic seizure':ti,kw,ab OR 'ischemic cerebral infarction':ti,kw,ab OR 'ischemic seizure':ti,kw,ab OR 'stroke*':ti,kw,ab | 600846 | 2024/9/30 |
| #1 | 'cerebrovascular accident'/exp | 467806 | 2024/9/30 |

**Cochrane**

Date Run:	30/09/2024 11:36:22

Comment:

ID	Search	Hits

#1	MeSH descriptor: [Stroke] explode all trees	17883

#2	(‘brain accident’ OR ‘brain attack’ OR ‘brain blood flow disturbance’ OR ‘brain insult*’ OR ‘brain vascular accident*’ OR ‘cerebral hemorrhage’ OR ‘cerebral infarction’ OR ‘cerebral insult’ OR ‘cerebral vascular accident’ OR ‘cerebral vascular insufficiency’ OR ‘cerebro vascular accident’ OR ‘cerebrovascular accident*’ OR ‘cerebrovascular arrest’ OR ‘cerebrovascular failure’ OR ‘cerebrovascular injury’ OR ‘cerebrovascular insufficiency’ OR ‘cerebrovascular insult’ OR ‘cerebrum vascular accident’ OR ‘CVA*’ OR ‘Hemorrhagic cerebral infarction’ OR ‘insultus cerebralis’ OR ‘ischaemic seizure’ OR ‘Ischemic cerebral infarction’ OR ‘ischemic seizure’ OR ‘stroke*’ ):ti,kw,ab	87021

#3	#1 OR #2	87127

#4	MeSH descriptor: [Sleep Initiation and Maintenance Disorders] explode all trees	3851

#5	MeSH descriptor: [Wakefulness] explode all trees	1364

#6	(‘agrypnia’ OR ‘awake*’ OR ‘DIMS’ OR ‘disorder* of sleep initiation and maintenance’ OR ‘Disorders of Initiating and Maintaining Sleep’ OR ‘Early Awakening’ OR ‘hyposomnia*’ OR ‘insomnia*’ OR ‘Sleep disorders’ OR ‘sleep initiation and maintenance disorder*’ OR ‘Sleep Initiation Dysfunction*’ OR ‘sleeplessness’ OR ‘wake up threshold’ OR ‘wakefullness’ OR ‘wakefulness*’ OR ‘waking’ OR ‘insomnia’ OR ‘wakefulness’):ti,kw,ab	36493

#7	#4 OR #5 OR #6	36493

#8	MeSH descriptor: [Acupuncture] explode all trees	224

#9	MeSH descriptor: [Electroacupuncture] explode all trees	1184

#10	MeSH descriptor: [Auriculotherapy] explode all trees	331

#11	(‘ACET’ OR ‘acupoint catgut embedding therapy’ OR ‘acupuncture*’ OR ‘auricular needling’ OR ‘auriculoacupuncture’ OR ‘Auriculotherap*’ OR ‘burnt needle therapy’ OR ‘catgut embedding’ OR ‘catgut implantation’ OR ‘ear point ‘ OR ‘Electroacupuncture’ OR ‘fire needle therapy’ OR ‘fire needling’ OR ‘imbedding needle’ OR ‘needle knife’ OR ‘needle punching ‘ OR ‘Pharmacopuncture’ OR ‘point embedding’ OR ‘shonishin’ OR ‘thermoacupuncture’ OR ‘warm needl*’ OR ‘warming’):ti,kw,ab	26254

#12	#8 OR #9 OR #10 OR #11	26254

#13	(‘random*’ OR ‘RCT’ OR ‘randomized controlled trial’ OR ‘control*’ OR

‘placebo’):ti,kw,ab	1711911

#14	#3 AND #7 AND #12 AND #13	78

**WOS**

| Search Query | Database | Results | Date Run |
| --- | --- | --- | --- |
| (brain accident) OR (brain attack) OR (brain blood flow disturbance) OR (brain insult*) OR (brain vascular accident*) OR (cerebral hemorrhage) OR (cerebral infarction) OR (cerebral insult) OR (cerebral vascular accident) OR (cerebral vascular insufficiency) OR (cerebro vascular accident) OR (cerebrovascular accident*) OR (cerebrovascular arrest) OR (cerebrovascular failure) OR (cerebrovascular injury) OR (cerebrovascular insufficiency) OR (cerebrovascular insult) OR (cerebrum vascular accident) OR (CVA*) OR (Hemorrhagic cerebral infarction) OR (insultus cerebralis) OR (ischaemic seizure) OR (Ischemic cerebral infarction) OR (ischemic seizure) OR (stroke*) (Topic) | Web of Science Core Collection | 329151 | Mon Sep 30 2024 12:04:27 GMT+0800 (GMT+08:00) |
| TS=((agrypnia) OR (awake*) OR (DIMS ) OR (disorder* of sleep initiation and maintenance) OR (Disorders of Initiating and Maintaining Sleep) OR (Early Awakening) OR (hyposomnia*) OR (insomnia*) OR (Sleep disorders) OR (sleep initiation and maintenance disorder*) OR (Sleep Initiation Dysfunction*) OR (sleeplessness) OR (wake up threshold) OR (wakefullness) OR (wakefulness*) OR (waking) OR (insomnia) OR (wakefulness)) | Web of Science Core Collection | 144048 | Mon Sep 30 2024 12:10:46 GMT+0800 (GMT+08:00) |
| ALL=((ACET) OR (acupoint catgut embedding therapy) OR (acupuncture*) OR (auricular needling) OR (auriculoacupuncture) OR (Auriculotherap*) OR (burnt needle therapy) OR (catgut embedding) OR (catgut implantation) OR (ear point ) OR (Electroacupuncture) OR (fire needle therapy) OR (fire needling) OR (imbedding needle) OR (needle knife) OR (needle punching ) OR (Pharmacopuncture) OR (point embedding) OR (shonishin) OR (thermoacupuncture) OR (warm needl*) OR (warming)) | Web of Science Core Collection | 227736 | Mon Sep 30 2024 12:11:01 GMT+0800 (GMT+08:00) |
| ALL=((random*) OR (RCT) OR (randomized controlled trial) OR (control*) OR (placebo)) | Web of Science Core Collection | 5146531 | Mon Sep 30 2024 12:11:19 GMT+0800 (GMT+08:00) |
| #4 AND #3 AND #2 AND #1 | Web of Science Core Collection | 54 | Mon Sep 30 2024 12:11:24 GMT+0800 (GMT+08:00) |
