# Supplementary material for: Effectiveness of acupuncture and related therapies for post-stroke insomnia: A systematic review and Bayesian network analysis
Source: Medicine (Baltimore). 2026 Aug 7;105(32):e50081. doi: 10.1097/MD.0000000000050081 (PMC13456779; doi:10.1097/MD.0000000000050081)
Supplement: Supplementary file 2 [file medi-105-e50081-s004.docx]

**Table S4. Summary Table of Basic Characteristics of Included Studies**

| ID | Author | Year | N(M/F) | Age | Intervention | Intervention period | Outcomes |
| --- | --- | --- | --- | --- | --- | --- | --- |
| 1 | Hao JB | 2016 | 27(13/14) | 57.71±8.25 | WA | 4w | PSQI |
|  |  |  | 27(12/15) | 58.02±7.96 | Med |  |  |
| 2 | Qin HJ | 2019 | 35(20/15) | 52.5±5.3 | Acu+Med | 4w | PSQI |
|  |  |  | 35(22/13) | 51.8±5.1 | Med |  |  |
| 3 | Wu Z | 2020 | 38(16/22) | 58.22±3.42 | Acu+Med | 4w | PSQI |
|  |  |  | 38(15/23) | 59.31±3.51 | Med |  |  |
| 4 | Feng JF | 2016 | 30(17/13) | 52.23±7.29 | Acu | 10d | PSQI |
|  |  |  | 30(14/16) | 55.20±6.66 | Med |  |  |
| 5 | Li KB | 2022 | 35(16/19) | 60.7 | Acu+AT | 2w | PSQI |
|  |  |  | 35(18/17) | 58.8 | Med |  |  |
| 6 | Jiang YY | 2018 | 29(18/11) | 58.62±8.58 | Acu | 3w | PSQI |
|  |  |  | 30(16/14) | 54.77±9.25 | Acu+Electro |  |  |
| 7 | Wang RY | 2023 | 16(7/9) | 61.13±6.74 | Electro | 3w | PSQI |
|  |  |  | 15(8/7) | 61.33±3.99 | CT |  |  |
| 8 | Sun YN | 2023 | 41(28/13) | 61.50±4.00 | Acu+Med | 6w | PSQI |
|  |  |  | 35(24/11) | 62.00±3.85 | Med |  |  |
| 9 | Ye FW | 2011 | 30(16/14) | 62.48±3.72 | Acu | 4w | PSQI |
|  |  |  | 30(17/13) | 62.40±4.88 | Med |  |  |
| 10 | Mai DY | 2022 | 31(14/17) | 60.22±9.37 | Acu+Med | 4w | PSQI |
|  |  |  | 31(15/16) | 59.45±8.36 | Med |  |  |
| 11 | Gou YH | 2015 | 30 | - | Acu | 2w | PSQI |
|  |  |  | 30 | - | Med |  |  |
| 12 | Gou YH | 2014 | 30 | - | Acu | - | PSQI |
|  |  |  | 30 | - | Med |  |  |
| 13 | Ma XM | 2016 | 40(24/16) | 61.88±5.16 | Acu | 4w | PSQI |
|  |  |  | 40(20/20) | 63.70±4.94 | Med |  |  |
| 14 | Wang Y | 2011 | 40(21/19) | 61.5±2.1 | Acu | 4w | PSQI |
|  |  |  | 38(20/18) | 63.1±2.5 | Med |  |  |
| 15 | Chen N | 2023 | 23(12/11) | 57.69±7.45 | Acu | 4w | PSQI |
|  |  |  | 22(12/10) | 58.74±7.56 | Med |  |  |
| 16 | Li CY | 2018 | 29 | 55.52±10.01 | Acu | 2w | PSQI |
|  |  |  | 27 | 57.19±9.92 | Med |  |  |
| 17 | Liu N | 2017 | 36(20/16) | 52.31±8.26 | AT+Med | 4w | PSQI |
|  |  |  | 36(24/12) | 52.08±6.19 | Med |  |  |
| 18 | Xing YS | 2014 | 32(17/15) | 55.4±6.4 | AT | 2w | PSQI |
|  |  |  | 32(16/16) | 54.8±7.2 | Med |  |  |
| 19 | Zhan DM | 2021 | 44(27/17) | 65.22±9.94 | Electro+Med | 4w | PSQI |
|  |  |  | 43(25/18) | 65.47±9.31 | Med |  |  |
| 20 | Su WJ | 2023 | 30(24/6) | 46.77±8.50 | AT+Med | 2w | PSQI |
|  |  |  | 30(20/10) | 50.93±8.97 | Med |  |  |
| 21 | Su WJ | 2021 | 30(17/13) | 56.30±9.09 | WA | 4w | PSQI |
|  |  |  | 30(20/10) | 56.43±9.01 | Acu |  |  |
| 22 | Zhuang XQ | 2022 | 23(14/9) | 63.87±3.03 | Acu | 4w | PSQI |
|  |  |  | 23(12/11) | 63.46±2.79 | Med |  |  |
| 23 | Tang B | 2024 | 54(30/24) | 70.5±6.5 | WA+Med | 5w | PSQI |
|  |  |  | 54(28/26) | 70.8±6.7 | Med |  |  |
| 24 | Li MX | 2020 | 25(12/13) | 69.6±17.2 | Electro | 30d | PSQI |
|  |  |  | 25(11/14) | 68.8±14.9 | Med |  |  |
| 25 | Zhang Y | 2020 | 44(25/19) | 67.48±5.26 | Acu+Med | 2w | PSQI |
|  |  |  | 44(24/20) | 67.25±5.20 | Med |  |  |
| 26 | Fu L | 2016 | 40(23/17) | 61±9 | Acu | 4w | PSQI |
|  |  |  | 40(22/18) | 63±9 | Med |  |  |
| 27 | An F | 2023 | 43(23/20) | 60±7 | Acu | 4w | PSQI |
|  |  |  | 45(25/20) | 61±8 | Med |  |  |
| 28 | Zou YL | 2020 | 30(16/14) | 57.2±12.72 | Acu | 3w | PSQI |
|  |  |  | 30(17/13) | 61.23±10.85 | Med |  |  |
| 29 | Peng TZ | 2023 | 30(18/12) | 65.76±8.62 | Acu | 2w | PSQI |
|  |  |  | 30(19/11) | 66.49±8.37 | Med |  |  |
| 30 | Luo LH | 2020 | 51(23/28) | 55.78±6.14 | Acu | 30d | PSQI |
|  |  |  | 51(22/29) | 55.65±6.22 | Med |  |  |
| 31 | Shang XN | 2024 | 36(19/17) | 60.01±5.00 | Acu | 1w | PSQI |
|  |  |  | 36(20/16) | 59.98±5.01 | Med |  |  |
| 32 | Guo YG | 2020 | 45(25/20) | 59.96±8.74 | Acu+AT | 20d | PSQI |
|  |  |  | 45(24/21) | 61.56±8.98 | Med |  |  |
| 33 | Chen YM | 2022 | 49(26/23) | 60.78±5.24 | Acu+Med | 1m | PSQI |
|  |  |  | 49(28/21) | 61.03±5.01 | Med |  |  |
| 34 | Liu X | 2024 | 48(25/23) | 54.86±5.91 | Acu+Med | 1m | PSQI |
|  |  |  | 47(26/21) | 54.92±6.37 | Med |  |  |
| 35 | Li MX | 2020 | 50(30/20) | 62.35±11.82 | Acu+Med | 1m | PSQI |
|  |  |  | 50(28/22) | 61.90±10.23 | Med |  |  |
| 36 | Chen JL | 2023 | 35(15/20) | 63.91±8.13 | Acu+AT | 4w | PSQI |
|  |  |  | 36(19/17) | 63.11±8.51 | Med |  |  |
| 37 | Li JX | 2024 | 30(19/11) | 63.75±3.42 | Acu | 4w | PSQI |
|  |  |  | 30(18/12) | 65.74±3.45 | Med |  |  |
| 38 | Lin ZB | 2023 | 27(15/12) | 48.25±7.81 | Acu+Med | 4w | PSQI |
|  |  |  | 27(14/13) | 50.05±6.67 | Med |  |  |
| 39 | Zhu H | 2024 | 33(20/13) | 68.42±6.31 | Acu | 2w | PSQI |
|  |  |  | 33(21/12) | 68.06±5.52 | Med |  |  |
| 40 | Jiao LN | 2021 | 29(15/14) | 66.5±7.8 | Acu | 30d | PSQI |
|  |  |  | 29(16/13) | 64.8±8.1 | Med |  |  |
| 41 | Yang HQ | 2024 | 64(31/33) | 67.29±1.52 | WA+Med | 2w | PSQI |
|  |  |  | 64(29/35) | 68.48±2.11 | Med |  |  |
| 42 | Liu L | 2020 | 30(12/18) | 60.7±11.6 | WA+AT | 4w | PSQI |
|  |  |  | 30(15/15) | 59.9±11.0 | Med |  |  |
| 43 | Su JY | 2022 | 37(22/15) | 59.05±10.57 | Acu+Med | 4w | PSQI |
|  |  |  | 38(16/22) | 60.21±10.51 | Med |  |  |
| 44 | Mi JP | 2009 | 40(27/13) | 65.28±10.549 | Acu | 4w | PSQI |
|  |  |  | 40(24/16) | 63.11±11.961 | Med |  |  |
| 45 | Liu JH | 2022 | 33(16/17) | 65.44±7.80 | Acu | 4w | PSQI |
|  |  |  | 33(18/15) | 65.16±7.84 | Med |  |  |
| 46 | Xu KX | 2023 | 41(22/19) | 66.43±5.75 | Acu+Med | 4w | PSQI |
|  |  |  | 41(25/16) | 65.42±5.46 | Med |  |  |
| 47 | Huang SM | 2021 | 40(22/18) | 60.6±3.8 | PE | 4w | PSQI |
|  |  |  | 40(21/19) | 60.5±3.9 | Med |  |  |
| 48 | Chen XD | 2017 | 60(32/28) | 62.4±2.5 | Acu | 4w | PSQI |
|  |  |  | 60(35/25) | 62.2±2.2 | CT |  |  |
| 49 | Guo SS | 2018 | 30(16/14) | 65.233±11.285 | Acu+Med | 2w | PSQI |
|  |  |  | 30(17/13) | 63.500±10.925 | Med |  |  |
| 50 | Lu YY | 2008 | 25(14/11) | 61.48±3.72 | Acu | 4w | PSQI |
|  |  |  | 25(15/10) | 62.40±4.88 | Med |  |  |
| 51 | Zhang HJ | 2022 | 50 | 67.38±2.98 | AT | 4w | PSQI |
|  |  |  | 47 | 67.62±3.60 | Med |  |  |
| 52 | Liu JX | 2017 | 35(21/14) | 62.08±7.82 | Acu | 4w | PSQI |
|  |  |  | 35(20/15) | 63.02±7.79 | Med |  |  |
| 53 | Zhou XP | 2022 | 39(20/19) | 69.21±2.02 | Acu+Med | 4w | PSQI |
|  |  |  | 39(21/18) | 68.11±1.47 | Med |  |  |
| 54 | Jia RZ | 2010 | 30(16/14) | 62.5±6.4 | Acu | 4w | PSQI |
|  |  |  | 30(13/17) | 63.1±7.0 | Med |  |  |
| 55 | Yang L | 2020 | 68(23/45) | 68.69±10.96 | Acu+AT | 20d | PSQI |
|  |  |  | 68(33/35) | 68.66±10.96 | Acu+AT+Med |  |  |
|  |  |  | 68(27/41) | 67.19±10.77 | Med |  |  |
| 56 | Wang HT | 2020 | 30(14/16) | 65±9 | Acu | 4w | PSQI |
|  |  |  | 30(15/15) | 67±8 | Med |  |  |
| 57 | Tan JY | 2019 | 30(16/14) | 64.87±8.98 | Acu+Med | 2w | PSQI |
|  |  |  | 30(17/13) | 62.57±7.38 | Med |  |  |
| 58 | He FL | 2020 | 30 | - | Acu | 2w | PSQI |
|  |  |  | 30 | - | Acu+Med |  |  |
|  |  |  | 30 | - | Med |  |  |
| 59 | Zhang YY | 2023 | 40(19/21) | 68±8 | Acu+AT | 6w | PSQI |
|  |  |  | 40(17/23) | 68±8 | Med |  |  |
| 60 | Liu CP | 2022 | 40(15/25) | 68.14±5.37 | Acu+Med | 1m | PSQI |
|  |  |  | 40(13/27) | 68.63±5.58 | Med |  |  |
| 61 | Sun YZ | 2011 | 30(14/16) | 40±15 | Acu+AT | 20d | PSQI |
|  |  |  | 30(15/15) | 40±16 | Med |  |  |
| 62 | Lao XT | 2018 | 45(22/23) | 69.2±9.6 | Acu+AT | 20d | PSQI |
|  |  |  | 45(21/24) | 68.5±10.2 | Med |  |  |
| 63 | Li J | 2019 | 55(33/22) | 51.58±4.12 | Acu+AT | 20d | PSQI |
|  |  |  | 55(32/23) | 51.32±4.19 | Med |  |  |
| 64 | Yang J | 2015 | 30(16/14) | 68.00±10.082 | Acu+AT | 20d | PSQI |
|  |  |  | 30(13/17) | 67.93±11.626 | Acu+AT+Med |  |  |
|  |  |  | 30(12/18) | 63.63±11.128 | Med |  |  |
| 65 | Zhang HY | 2015 | 30(16/14) | 69.37±9.58 | Acu+AT | 20d | PSQI |
|  |  |  | 30(11/19) | 68.97±8.57 | Med |  |  |
| 66 | Zhao JY | 2016 | 47(20/27) | 67.15±12.375 | Acu+AT | 20d | PSQI |
|  |  |  | 53(25/28) | 69.08±12.318 | Acu+AT+Med |  |  |
|  |  |  | 50(19/31) | 64.42±12.281 | Med |  |  |
| 67 | Wang YP | 2018 | 76(40/36) | 64±6 | Acu+Med | 4w | PSQI |
|  |  |  | 76(37/39) | 64±6 | Med |  |  |
| 68 | Yu XJ | 2021 | 30(12/18) | 65.0±10.5 | Acu+AT | 4w | PSQI |
|  |  |  | 30(14/16) | 65.0±10.1 | Med |  |  |
| 69 | Zhang WM | 2012 | 40(24/16) | 58.7 | Acu | 4w | PSQI |
|  |  |  | 40(21/19) | 60.4 | Med |  |  |
| 70 | Ye CH | 2013 | 43(23/20) | 62.8±7.2 | Acu | 4w | PSQI |
|  |  |  | 42(20/22) | 67.3±8.3 | Med |  |  |
| 71 | Li TB | 2007 | 32(18/14) | 69.8±7.1 | Acu | 4w | PSQI |
|  |  |  | 32(17/15) | 67.3±8.3 | Med |  |  |
| 72 | Wang Y | 2004 | 34(22/12) | - | Acu+Electro+AT | 4w | PSQI |
|  |  |  | 30(17/13) | - | Med |  |  |
| 73 | Liu JH | 2006 | 32(15/17) | 69.9±6.9 | Acu | 4w | PSQI |
|  |  |  | 30(15/15) | 67.5±8.2 | Med |  |  |
| 74 | Lan HQ | 2021 | 28(18/10) | 58.26±4.62 | Acu+Med | 4w | PSQI |
|  |  |  | 28(17/11) | 58.35±4.25 | Med |  |  |
| 75 | Zhu C | 2014 | 52(30/22) | 75.2±1.3 | Acu | 4w | PSQI |
|  |  |  | 44(26/18) | 74.3±2.9 | Med |  |  |
| 76 | Wang MC | 2023 | 30(18/12) | 64.89±7.05 | Acu+Med | 4w | PSQI |
|  |  |  | 35(19/16) | 64.46±9.56 | Med |  |  |
| 77 | Wen SD | 2023 | 35(21/14) | 61.41±2.22 | Acu | 4w | PSQI |
|  |  |  | 35(19/16) | 61.23±2.28 | Med |  |  |
| 78 | Zhang XL | 2021 | 33(21/12) | 54.35±9.04 | WA+Med | 2w | PSQI |
|  |  |  | 33(19/14) | 54.25±8.89 | Med |  |  |
| 79 | Wang WY | 2022 | 32(18/14) | 61.6±4.0 | Acu+AT | 2w | PSQI |
|  |  |  | 32(17/15) | 61.1±4.5 | Med |  |  |
| 80 | Zhang YY | 2020 | 39(23/16) | 52.48±4.13 | Acu+Med | 4w | PSQI |
|  |  |  | 39(21/18) | 51.63±3.54 | Med |  |  |
| 81 | Xu F | 2016 | 20(12/8) | 56.26±4.29 | Electro+AT | 4w | PSQI |
|  |  |  | 20(11/9) | 56.55±4.36 | Med |  |  |
| 82 | Cao Y | 2022 | 72(50/22) | 65.2±7.10 | Acu | 4w | PSQI |
|  |  |  | 72(49/23) | 63.6±8.25 | CT |  |  |
| 83 | Cai SM | 2020 | 43(22/21) | 63.37±10.42 | Acu | 8w | PSQI |
|  |  |  | 43(24/19) | 64.77±8.53 | Med |  |  |
| 84 | Zeng TT | 2022 | 30(13/17) | 63.44±3.67 | Acu+PE | 2w | PSQI |
|  |  |  | 30(14/16) | 62.41±3.52 | Acu |  |  |
| 85 | Liu Y | 2021 | 30(18/12) | 62.83±7.48 | Acu+Electro | 2w | PSQI |
|  |  |  | 30(16/14) | 62.60±7.04 | Acu |  |  |
| 86 | Tang LJ | 2023 | 49(28/21) | 48.6±10.7 | Acu+AT | 4w | PSQI |
|  |  |  | 49(26/23) | 49.2±11.6 | AT |  |  |
| 87 | Ye XY | 2023 | 34(14/20) | 62.88±6.759 | Acu+AT | 4w | PSQI |
|  |  |  | 32(11/21) | 63.13±6.559 | Acu |  |  |
| 88 | Chen CY | 2022 | 33(15/18) | 62.76±7.47 | Acu+Electro | 2w | PSQI |
|  |  |  | 34(18/16) | 62.82±8.70 | Acu |  |  |
| 89 | Sun X | 2024 | 50(29/21) | 58.30±5.12 | Electro | 4w | PSQI |
|  |  |  | 50(26/24) | 60.57±6.04 | Acu |  |  |
| 90 | Cao Y | 2020 | 43(27/16) | 68.4±5.1 | Acu | 4w | PSQI |
|  |  |  | 41(25/16) | 67.3±5.9 | CT |  |  |
| 91 | Cao Y | 2020 | 36(23/13) | 63±9 | Acu | 4w | PSQI |
|  |  |  | 36(30/6) | 63±6 | CT |  |  |
| 92 | Huang HX | 2019 | 29(18/11) | 64.9±3.7 | AT | 80d | PSQI |
|  |  |  | 29(19/10) | 65.6±4.4 | CT |  |  |
| 93 | Wu MJ | 2022 | 35(19/16) | 58.51±7.84 | Acu+AT | 4w | PSQI |
|  |  |  | 35(20/15) | 59.74±10.44 | Acu |  |  |
| 94 | Shao ZJ | 2023 | 30(14/16) | 68.61±11.23 | Acu+Med | 2w | PSQI |
|  |  |  | 30(16/14) | 67.56±12.78 | Med |  |  |
| 95 | Sun YZ | 2013 | 30(12/18) | 57±9 | Acu+PE | 8w | PSQI |
|  |  |  | 30(15/15) | 55±10 | Acu+AT |  |  |
| 96 | Ji HJ | 2022 | 10(6/4) | 56.78±5.03 | AT | 2w | PSQI |
|  |  |  | 10(5/5) | 56.82±5.07 | CT |  |  |
| 97 | Li LL | 2013 | 36(24/12) | 65.8±12.1 | AT | 3w | PSQI |
|  |  |  | 34(22/12) | 62.3±17.6 | CT |  |  |
| 98 | Ding WL | 2023 | 50(31/19) | 59.42±2.65 | Acu+AT | 2w | PSQI |
|  |  |  | 50(33/17) | 60.45±2.78 | AT |  |  |
| 99 | Li X | 2019 | 27(19/8) | 64.00±8.11 | AT | 4w | PSQI |
|  |  |  | 28(17/11) | 60.75±9.59 | CT |  |  |
| 100 | Liu XH | 2022 | 51(21/30) | 56.23±8.56 | Acu+AT | 4w | PSQI |
|  |  |  | 47(16/31) | 55.98±8.42 | AT |  |  |
| 101 | Liu X | 2021 | 30(16/14) | 61.9±8.31 | Acu+AT | 2w | PSQI |
|  |  |  | 30(18/12) | 62.3±7.98 | Acu+Med |  |  |
| 102 | Li ZY | 2021 | 40(27/13) | 66.7±4.6 | AT | 48d | PSQI |
|  |  |  | 40(25/15) | 67.1±4.5 | CT |  |  |
| 103 | Sun Y | 2020 | 44(31/13) | 56.17±6.25 | Acu+AT | 2m | PSQI |
|  |  |  | 44(28/16) | 56.39±6.09 | CT |  |  |
| 104 | Yin CY | 2015 | 33(22/11) | 63.7±11.2 | AT | 4w | PSQI |
|  |  |  | 35(20/15) | 61.5±12.0 | CT |  |  |
| 105 | Jin X | 2021 | 30(14/16) | 59.3 | Acu | 2w | PSQI |
|  |  |  | 30(12/18) | 59.7 | Med |  |  |
| 106 | Fan YY | 2019 | 41(22/19) | 63.1±8.4 | Acu+AT | 4w | PSQI |
|  |  |  | 41(23/18) | 62.8±8.9 | Acu |  |  |
| 107 | Zhang YY | 2020 | 34(20/14) | 55.3 | WA | 6w | PSQI |
|  |  |  | 34(22/12) | 56.4 | Med |  |  |
