# Supplementary material for: Effectiveness of acupuncture and related therapies for post-stroke insomnia: A systematic review and Bayesian network analysis
Source: Medicine (Baltimore). 2026 Aug 7;105(32):e50081. doi: 10.1097/MD.0000000000050081 (PMC13456779; doi:10.1097/MD.0000000000050081)
Supplement: Supplementary file 3 [file medi-105-e50081-s002.doc]

**Table S2. Summary Table of GRADE Certainty of Evidence Assessments**

| Comparison | Direct evidence | | Indirect evidence | | NetworkMeta-Analysis | |
| --- | --- | --- | --- | --- | --- | --- |
|  | SMD(95%CI) | Certainty of evidence | OR(95%CI) | Certainty of evidence | OR(95%CI) | Certainty of evidence |
| Acu vs Med | -0.87(-0.97, -0.78) | ⊕○○○ | − | − | -2.45(-3.11, -1.79) | ⊕○○○ |
| Acu+Electro vs Acu | -0.68(-1.05, -0.32) | ⊕○○○ | − | − | -2.00 (-4.86, 0.87) | ⊕○○○ |
| Acu vs CT | -1.06(-1.29, -0.83) | ⊕○○○ | − | − | -3.26 (-4.65, -1.88) | ⊕○○○ |
| Electro vs Acu | -0.35(-0.67, -0.04) | ⊕⊕○○ | − | − | -0.33(-2.31, 1.65) | ⊕⊕○○ |
| WA vs Acu | -10.08(-12.01, -8.14) | ⊕⊕○○ | − | − | -0.87 (-3.59, 1.88) | ⊕⊕○○ |
| Acu+Med vs Acu | 0.00(-0.51, 0.51) | ⊕○○○ | − | − | 0.08 (-1.01, 1.17) | ⊕○○○ |
| Acu+PE vs Acu | -1.64(-2.23, -1.05) | ⊕⊕○○ | − | − | -2.27 (-4.61, 0.05) | ⊕⊕○○ |
| Acu+AT vs Acu | -0.91(-1.19, -0.63) | ⊕⊕○○ | − | − | -0.83 (-1.84, 0.17) | ⊕⊕○○ |
| WA+AT vs Acu | − | − | 0.32(0.06, 0.59) | ⊕○○○ | -0.32 (-4.93, 4.36) | ⊕○○○ |
| WA+Med vs Acu | − | − | 0.15(-0.01, 0.32) | ⊕○○○ | 0.17 (-2.21, 2.55) | ⊕○○○ |
| AT+Med vs Acu | − | − | 0.28(0.03, 0.54) | ⊕○○○ | 0.65 (-2.34, 3.67) | ⊕○○○ |
| Acu+Electro+AT vs Acu | − | − | -0.25(-0.56, 0.08) | ⊕○○○ | -3.24 (-6.56, 0.13) | ⊕○○○ |
| Electro+Med vs Acu | − | − | 0.19(-0.14, 0.53) | ⊕○○○ | 0.61 (-3.31, 4.55) | ⊕○○○ |
| Acu+AT+Med vs Acu | − | − | 0.62(0.49, 0.76) | ⊕○○○ | 1.64 (-0.46, 3.71) | ⊕○○○ |
| AT vs Acu | − | − | 0.1(-0.12, 0.33) | ⊕○○○ | 1.16 (-0.33, 2.65) | ⊕○○○ |
| WA vs Med | -0.09(-0.63, 0.44) | ⊕○○○ | − | − | -3.33 (-6.06, -0.60) | ⊕○○○ |
| WA vs Electro | − | − | -9.73 (-11.34, -8.1 ) | ⊕⊕○○ | -0.54 (-3.86, 2.81) | ⊕⊕○○ |
| WA vs CT | − | − | -0.28(-0.68, 0.12) | ⊕○○○ | -4.13 (-7.15, -1.07) | ⊕○○○ |
| WA vs Acu+Electro | − | − | -9.4(-10.96, -7.82) | ⊕○○○ | -0.54 (-3.86, 2.81) | ⊕○○○ |
| WA vs Acu+PE | − | − | -8.44(-9.78, -7.09) | ⊕⊕○○ | 1.4 (-2.14, 4.97) | ⊕⊕○○ |
| WA vs Acu+Med | − | − | -10.08(-11.5, -8.65) | ⊕○○○ | -0.96 (-3.82, 1.91) | ⊕○○○ |
| WA vs Acu+AT | − | − | -9.17(-10.82, -7.51) | ⊕⊕○○ | -0.04 (-2.9, 2.82) | ⊕⊕○○ |
| WA vs AT | − | − | 0.68(0.46, 0.89) | ⊕○○○ | -2.03 (-5.11, 1.09) | ⊕○○○ |
| WA vs Acu+AT+Med | − | − | 0.16(-0.15, 0.46) | ⊕○○○ | -2.51 (-5.86, 0.88) | ⊕○○○ |
| WA vs Electro+Med | − | − | 0.59(0.48, 0.69) | ⊕○○○ | -1.49 (-6.27, 3.27) | ⊕○○○ |
| WA vs Acu+Electro+AT | − | − | 1.03(0.9, 1.14) | ⊕○○○ | 2.36 (-1.9, 6.64) | ⊕○○○ |
| WA vs AT+Med | − | − | 0.5(0.31, 0.68) | ⊕○○○ | -1.53 (-5.52, 2.5) | ⊕○○○ |
| WA vs WA+Med | − | − | 0.63(0.35, 0.9) | ⊕○○○ | -1.03 (-4.60, 2.53) | ⊕○○○ |
| WA vs WA+AT | − | − | 0.46(0.28, 0.63) | ⊕○○○ | -0.55 (-5.91, 4.79) | ⊕○○○ |
| Electro vs Med | -0.35(-0.91, 0.21) | ⊕○○○ | − | − | -2.78(-4.80, -0.79) | ⊕○○○ |
| Electro vs CT | -2.80(-3.82, -1.77) | ⊕⊕○○ | − | − | -3.6 (-5.78, -1.4) | ⊕⊕○○ |
| Electro vs Acu+Electro | − | − | 0.33(0.38, 0.28) | ⊕○○○ | 1.67 (-1.8, 5.14) | ⊕○○○ |
| Electro vs Acu+PE | − | − | 1.29(1.56, 1.01) | ⊕⊕○○ | 1.94 (-1.08, 4.95) | ⊕⊕○○ |
| Electro vs Acu+Med | − | − | -0.35(-0.16, -0.55) | ⊕○○○ | -0.41 (-2.6, 1.75) | ⊕○○○ |
| Electro vs Acu+AT | − | − | 0.56(0.52, 0.59) | ⊕⊕○○ | 0.5 (-1.65, 2.63) | ⊕⊕○○ |
| Electro vs AT | − | − | -2.17(-3.02, -1.32) | ⊕⊕○○ | -1.5 (-3.83, 0.84) | ⊕⊕○○ |
| Electro vs Acu+AT+Med | − | − | -0.1(-0.43, 0.23) | ⊕○○○ | -1.96 (-4.77, 0.83) | ⊕○○○ |
| Electro vs Electro+Med | − | − | 0.33(0.2, 0.46) | ⊕○○○ | -0.94 (-5.34, 3.40) | ⊕○○○ |
| Electro vs Acu+Electro+AT | − | − | 0.77(0.62, 0.91) | ⊕○○○ | 2.9 (-0.95, 6.70) | ⊕○○○ |
| Electro vs AT+Med | − | − | 0.24(0.03, 0.45) | ⊕○○○ | -0.99 (-4.55, 2.56) | ⊕○○○ |
| Electro vs WA+Med | − | − | 0.37(0.07, 0.67) | ⊕○○○ | -0.50 (-3.55, 2.55) | ⊕○○○ |
| Electro vs WA+AT | − | − | 0.2(0, 0.4) | ⊕○○○ | -0.02 (-5.04, 4.97) | ⊕○○○ |
| AT vs Med | -0.77(-1.09, -0.45) | ⊕○○○ | − | − | -1.29 (-2.78, 0.178) | ⊕○○○ |
| Acu+AT vs AT | -1.24(-1.49, -0.99) | ⊕⊕○○ | − | − | -1.99(-3.48, -0.51) | ⊕⊕○○ |
| AT vs CT | -0.63(-0.80, -0.45) | ⊕⊕○○ | − | − | -2.10 (-3.47, -0.74) | ⊕⊕○○ |
| AT vs Acu+AT+Med | − | − | -0.52(-0.61, -0.43) | ⊕○○○ | -0.48 (-2.89, 1.94) | ⊕○○○ |
| AT vs Electro+Med | − | − | -0.09(0.02, -0.2) | ⊕○○○ | 0.55 (-3.63, 4.66) | ⊕○○○ |
| AT vs Acu+Electro+AT | − | − | 0.35(0.44, 0.25) | ⊕○○○ | 4.41 (0.81, 8.00) | ⊕○○○ |
| AT vs AT+Med | − | − | -0.18(-0.15, -0.21) | ⊕○○○ | 0.51 (-2.78, 3.81) | ⊕○○○ |
| AT vs WA+Med | − | − | -0.05(-0.11, 0.01) | ⊕○○○ | 0.99 (-1.71, 3.73) | ⊕○○○ |
| AT vs WA+AT | − | − | -0.22(-0.18, -0.26) | ⊕○○○ | 1.48(-3.36, 6.24) | ⊕○○○ |
| AT vs Acu+Electro | − | − | 0.78(0.93, 0.65) | ⊕○○○ | 3.16 (-0.07, 6.38) | ⊕○○○ |
| AT vs Acu+PE | − | − | 0.23(0.48, -0.02) | ⊕○○○ | 3.43 (0.77, 6.1) | ⊕○○○ |
| AT vs Acu+Med | − | − | 0.15(-0.07, 0.37) | ⊕○○○ | 1.08 (-0.63, 2.78) | ⊕○○○ |
| Acu+PE vs Med | -1.00(-1.57, -0.43) | ⊕⊕○○ | − | − | -4.72 (-7.05, -2.42) | ⊕⊕○○ |
| Acu+PE vs Acu+AT | -0.62(-1.14, -0.10) | ⊕⊕○○ | − | − | -1.44 (-3.81, 0.92) | ⊕⊕○○ |
| Acu+PE vs Acu+Med | − | − | -1.3(-1.3, -1.31) | ⊕⊕○○ | -2.35 (-4.83, 0.11) | ⊕⊕○○ |
| Acu+PE vs Acu+AT+Med | − | − | -0.75(-1.09, -0.41) | ⊕⊕○○ | -3.9 (-6.93, -0.91) | ⊕⊕○○ |
| Acu+PE vs Electro+Med | − | − | -0.32(-0.46, -0.18) | ⊕○○○ | -2.88 (-7.43, 1.58) | ⊕○○○ |
| Acu+PE vs Acu+Electro+AT | − | − | 0.12(-0.04, 0.27) | ⊕⊕○○ | 0.96 (-3.04, 4.97) | ⊕⊕○○ |
| Acu+PE vs AT+Med | − | − | -0.41(-0.63, -0.19) | ⊕⊕○○ | -2.93 (-6.67, 0.80) | ⊕⊕○○ |
| Acu+PE vs WA+Med | − | − | -0.28(-0.59, 0.03) | ⊕⊕○○ | -2.44 (-5.68, 0.79) | ⊕⊕○○ |
| Acu+PE vs WA+AT | − | − | -0.45(-0.66, -0.24) | ⊕⊕○○ | -1.95 (-7.16, 3.15) | ⊕⊕○○ |
| Acu+PE vs Acu+Electro | − | − | -0.96(-1.18, -0.73) | ⊕○○○ | -0.27 (-3.94, 3.42) | ⊕○○○ |
| Acu+PE vs CT | − | − | −4.09 (−2.78, −5.38) | ⊕⊕○○ | -5.53 (-8.18, -2.89) | ⊕⊕○○ |
| Acu+Med vs Med | -0.92(-1.02, -0.82) | ⊕○○○ | − | − | -2.37 (-3.26, -1.49) | ⊕○○○ |
| Acu+AT vs Acu+Med | -0.68(-1.21, -0.16) | ⊕⊕○○ | − | − | -0.92 (-2.14, 0.31) | ⊕⊕○○ |
| Acu+Med vs Acu+AT+Med | − | − | -0.67(-0.54, -0.8) | ⊕○○○ | -1.55 (-3.73, 0.65) | ⊕○○○ |
| Acu+Med vs Electro+Med | − | − | -0.24(0.09, -0.57) | ⊕○○○ | -0.54 (-4.51, 3.45) | ⊕○○○ |
| Acu+Med vs Acu+Electro+AT | − | − | 0.2(0.51, -0.12) | ⊕○○○ | 3.32 (-0.09, 6.7) | ⊕○○○ |
| Acu+Med vs AT+Med | − | − | -0.33(0.08, -0.58) | ⊕○○○ | -0.58 (-3.66, 2.50) | ⊕○○○ |
| Acu+Med vs WA+Med | − | − | -0.2(-0.04, -0.36) | ⊕○○○ | -0.08 (-2.54, 2.36) | ⊕○○○ |
| Acu+Med vs WA+AT | − | − | -0.37(-0.11, -0.63) | ⊕○○○ | 0.40 (-4.30, 5.05) | ⊕○○○ |
| Acu+Med vs Acu+Electro | − | − | 0.68(0.54, 0.83) | ⊕○○○ | 2.08 (-0.96, 5.16) | ⊕○○○ |
| Acu+Med vs CT | − | − | -1.11(-1.07, -1.14) | ⊕○○○ | -3.17 (-4.84, -1.51) | ⊕○○○ |
| Acu+AT vs Med | -1.01(-1.14, -0.87) | ⊕○○○ | − | − | -3.28 (-4.18, -2.40) | ⊕○○○ |
| Acu+AT vs CT | 0.20(-0.14, 0.54) | ⊕○○○ | − | − | -4.09 (-5.59, -2.60) | ⊕○○○ |
| Acu+AT vs Electro+Med | − | − | -0.33(-0.03, -0.62) | ⊕○○○ | -1.44 (-5.43, 2.51) | ⊕○○○ |
| Acu+AT vs Acu+Electro+AT | − | − | 0.11(0.39, -0.17) | ⊕○○○ | 2.41 (-1.00, 5.77) | ⊕○○○ |
| Acu+AT vs AT+Med | − | − | -0.42(-0.2, -0.63) | ⊕○○○ | -1.48 (-4.56, 1.58) | ⊕○○○ |
| Acu+AT vs WA+Med | − | − | -0.29(-0.16, -0.41) | ⊕○○○ | -1.00 (-3.46, 1.45) | ⊕○○○ |
| Acu+AT vs WA+AT | − | − | -0.46(-0.23, -0.68) | ⊕○○○ | -0.51 (-5.20, 4.12) | ⊕○○○ |
| Acu+AT vs Acu+Electro | − | − | -0.23(-0.14, -0.31) | ⊕○○○ | 1.18 (-1.87, 4.20) | ⊕○○○ |
| Acu+AT+Med vs Med | -0.25(-0.48, -0.02) | ⊕⊕○○ | − | − | -0.81 (-2.84, 1.19) | ⊕⊕○○ |
| Acu+AT+Med vs CT | − | − | -0.44(-0.53, -0.34) | ⊕○○○ | -1.62 (-4.03, 0.77) | ⊕○○○ |
| Acu+AT+Med vs Electro+Med | − | − | 0.43(0.63, 0.23) | ⊕⊕○○ | 1.02 (-3.35, 5.38) | ⊕⊕○○ |
| Acu+AT+Med vs Acu+Electro+AT | − | − | 0.87(1.05, 0.68) | ⊕⊕○○ | 4.88(1.01, 8.70) | ⊕⊕○○ |
| Acu+AT+Med vs AT+Med | − | − | 0.34(0.46, 0.22) | ⊕⊕○○ | 0.98 (-2.59, 4.50) | ⊕⊕○○ |
| Acu+AT+Med vs WA+Med | − | − | 0.47(0.5, 0.44) | ⊕⊕○○ | 1.47 (-1.58, 4.50) | ⊕⊕○○ |
| Acu+AT+Med vs WA+AT | − | − | 0.3(0.43, 0.17) | ⊕⊕○○ | 1.96 (-3.08, 6.95) | ⊕⊕○○ |
| Acu+AT+Med vs Acu+Electro | − | − | 1.3(1.54, 1.08) | ⊕○○○ | 3.63 (0.10, 7.21) | ⊕○○○ |
| Electro+Med vs Med | -0.68(-1.11, -0.25) | ⊕⊕○○ | − | − | -1.83 (-5.70, 2.05) | ⊕⊕○○ |
| Electro+Med vs CT | − | − | -0.87(-1.16, -0.57) | ⊕○○○ | -2.65 (-6.75, 1.53) | ⊕○○○ |
| Electro+Med vs Acu+Electro+AT | − | − | 0.44(0.42, 0.45) | ⊕⊕○○ | 3.85 (-1.22, 8.94) | ⊕⊕○○ |
| Electro+Med vs AT+Med | − | − | -0.09(-0.17, -0.01) | ⊕⊕○○ | -0.03 (-4.9, 4.82) | ⊕⊕○○ |
| Electro+Med vs WA+Med | − | − | 0.04(-0.13, 0.21) | ⊕⊕○○ | 0.46 (-4.03, 4.95) | ⊕⊕○○ |
| Electro+Med vs WA+AT | − | − | -0.13(-0.2, -0.06) | ⊕⊕○○ | 0.93 (-5.04, 6.95) | ⊕⊕○○ |
| Electro+Med vs Acu+Electro | − | − | 0.87(0.91, 0.85) | ⊕○○○ | 2.62 (-2.23, 7.51) | ⊕○○○ |
| AT+Med vs Med | -0.59(-0.94, -0.24) | ⊕⊕○○ | − | − | -1.79 (-4.72, 1.14) | ⊕⊕○○ |
| AT+Med vs CT | − | − | -0.78(-0.99, -0.56) | ⊕○○○ | -2.60 (-5.85, 0.66) | ⊕○○○ |
| AT+Med vs Acu+Electro+AT | − | − | 0.53(0.59, 0.46) | ⊕⊕○○ | 3.9 (-0.53, 8.27) | ⊕⊕○○ |
| AT+Med vs WA+Med | − | − | 0.13(0.04, 0.22) | ⊕⊕○○ | 0.49 (-3.20, 4.20) | ⊕⊕○○ |
| AT+Med vs WA+AT | − | − | -0.04(-0.03, -0.05) | ⊕⊕○○ | 0.98 (-4.51, 6.44) | ⊕⊕○○ |
| AT+Med vs Acu+Electro | − | − | 0.96(1.08, 0.86) | ⊕○○○ | 2.66 (-1.49, 6.81) | ⊕○○○ |
| WA+AT vs Med | -0.55(-0.91, -0.19) | ⊕⊕○○ | − | − | -2.78 (-7.34, 1.84) | ⊕⊕○○ |
| WA+AT vs CT | − | − | -0.74(-0.96, -0.51) | ⊕○○○ | -3.58 (-8.33, 1.26) | ⊕○○○ |
| WA+AT vs Acu+Electro+AT | − | − | 0.57(0.62, 0.51) | ⊕⊕○○ | 2.92 (-2.65, 8.56) | ⊕⊕○○ |
| WA+AT vs WA+Med | − | − | 0.17(0.07, 0.27) | ⊕⊕○○ | -0.48 (-5.57, 4.67) | ⊕⊕○○ |
| WA+AT vs Acu+Electro | − | − | 1(1.11, 0.91) | ⊕○○○ | 1.69 (-3.73, 7.15) | ⊕○○○ |
| WA+Med vs Med | -0.72(-0.98, -0.46) | ⊕⊕○○ | − | − | -2.29 (-4.56, 0) | ⊕⊕○○ |
| WA+Med vs CT | − | − | -0.91(-1.03, -0.78) | ⊕○○○ | -3.09 (-5.8, -0.41) | ⊕○○○ |
| WA+Med vs Acu+Electro+AT | − | − | 0.4(0.55, 0.24) | ⊕⊕○○ | 3.4 (-0.6, 7.37) | ⊕⊕○○ |
| WA+Med vs Acu+Electro | − | − | 0.83(1.04, 0.64) | ⊕○○○ | 2.16 (-1.54, 5.88) | ⊕○○○ |
| Acu+Electro vs Med | − | − | -1.55(-1.83, -1.29) | ⊕○○○ | -4.46 (-7.40, -1.52) | ⊕○○○ |
| Acu+Electro vs CT | − | − | -1.74(-1.88, -1.61) | ⊕○○○ | -5.25 (-8.44, -2.09) | ⊕○○○ |
| Acu+Electro vs Acu+Electro+AT | − | − | -0.43(-0.3, -0.59) | ⊕○○○ | 1.23 (-3.16, 5.65) | ⊕○○○ |
| Acu+Electro+AT vs Med | -1.12(-1.53, -0.70) | ⊕⊕○○ | − | − | -5.69 (-8.95, -2.40) | ⊕⊕○○ |
| Acu+Electro+AT vs CT | − | − | -1.31(-1.58, -1.02) | ⊕○○○ | -6.49 (-10.07, -2.89) | ⊕○○○ |
| CT vs Med | − | − | 0.19(0.05, 0.32) | ⊕○○○ | 0.80(-0.62, 2.22) | ⊕○○○ |
| Acu+AT+Med vs Acu+AT | 0.72(0.48, 0.96) | ⊕○○○ |  |  | 2.47 (0.44, 4.48) | ⊕○○○ |

⊕⊕○○ Low; ⊕○○○Very Low.
