# Supplementary material for: Effectiveness of acupuncture and related therapies for post-stroke insomnia: A systematic review and Bayesian network analysis
Source: Medicine (Baltimore). 2026 Aug 7;105(32):e50081. doi: 10.1097/MD.0000000000050081 (PMC13456779; doi:10.1097/MD.0000000000050081)
Supplement: Supplementary file 4 [file medi-105-e50081-s003.doc]

**Table S3. List of Excluded Studies and Reasons for Exclusion**

| **Year** | **Author** | **Title** | **Reason for Exclusion** |
| --- | --- | --- | --- |
| 2004 | Kim Y.S. | Intradermal acupuncture on shen-men and nei-kuan acupoints in patients with insomnia after stroke | No Data |
| 2009 | Lee S Y | Intradermal acupuncture on shen-men and nei-kuan acupoints improves insomnia in stroke patients by reducing the sympathetic nervous activity: a randomized clinical trial | Not meeting study aim |
| 2009 | Zhang JJ | Catgut lmplantation at the Treatment of insomnia in Patientswith Post-stroke Recovery Clinical Observation | No Data |
| 2010 | Ye FW | Clinical Observation of Acupuncture at Baihui (GV20) for Post-Stroke Insomnia: 30 Cases | No Data |
| 2011 | Wang LH | Efficacy Observation of Auricular Acupuncture for Post-Stroke Insomnia | No Data |
| 2012 | Chen XJ | Efficacy Evaluation of Treating Post-stroke Insomnia Majorly by Acupuncture at Four Insomnia Acupoints | Not meeting study aim |
| 2012 | Wei ZL | Clinical research of Jin three needle treatment ischemic onstroke in the acute phase of sleep disorders | Not meeting study aim |
| 2012 | Huang M | Effect Observation of Acupuncture-Moxibustion on Post-Stroke Sleep Disorder Patients | No Data |
| 2012 | Li G | Efficacy Observation of Acupuncture Combined with Estazolam for Post-Stroke Insomnia | No Data |
| 2012 | Wang GM | Clinical Observation of Acupuncture for Post-Stroke Insomnia in 32 Cases | No Data |
| 2012 | Wei YH | Efficacy Comparison for Sleep Disorders in Acute-Stage Stroke Patients | No Data |
| 2012 | Wu GY | Efficacy Analysis of Auricular Seed Pressing for Insomnia Improvement in Stroke Patients | No Data |
| 2012 | Wu XL | Syndrome-Differentiated Auricular Acupuncture for Post-Stroke Insomnia: 40 Case Studies | No Data |
| 2013 | Bi YZ | 20 Cases of Post-Stroke Sleep Disorders Treated with "Mian Sanzhen" | Not meeting study aim |
| 2013 | Sun LH | Cowherb seed seed ear beans to improve cerebral blood Tube accidental sleep disordersin patients with clinical observation | Not meeting study aim |
| 2013 | Xing H | Clinical observation on the therapeutic effect ofacupuncturing twelve yuan acupoints | Not meeting study aim |
| 2013 | Wang C | Clinical Experience of Auricular Seed Pressing in Elderly Stroke Patients with Insomnia | No Data |
| 2014 | Kui Y | Efficacy Evaluation of Changqi Tongluo Acupuncture for Post-Stroke Sleep Disorders | Not meeting study aim |
| 2014 | Shao ZJ | Clinical Observation of Insomnia of Ear Pressure Beans After Stroke Treated with TCM Syndrome Differentiation | Not meeting study aim |
| 2014 | Xie J | The Clinicai Study On Tongdu Tiaoshenacupuncture For The Treatment Ofsleep Disorder After Stroke | Not meeting study aim |
| 2014 | You L | Clinical Observation of Acupuncture for Post-Cerebral Infarction Insomnia | Not meeting study aim |
| 2014 | Lv XH | Acupuncture for Post-Stroke Depressive Insomnia | No Data |
| 2014 | Wang YJ | Effect of Auricular Seed Pressing on Sleep Status in Stroke Patients | No Data |
| 2014 | Zhao Y | Acupuncture-Moxibustion for Post-Stroke Insomnia | No Data |
| 2015 | Han XC | The Clinical Study of Post-stroke Sleep Disorders treatedwith Nazi-Method of Midnight-noon Ebb-flow | Not meeting study aim |
| 2015 | Hong XY | Observation on the Therapeutic Effectiveness of Changqi Tongluo Acupuncture for Post-Stroke Sleep Disorders | Not meeting study aim |
| 2015 | Li CH | Clinical Observation of Abdominal Acupuncture for Post-Stroke Insomnia | Not meeting study aim |
| 2015 | Tang Y | Clinical Observation of Acupuncture at "Three-Shen Points" (Sanshen Xue) for Post-Stroke Insomnia | Not meeting study aim |
| 2015 | Yang HL | Observation on the Effect of "Disease Combined with Syndrome”Acupuncture in reatingSleep Disorder of Liver-yin and Kidney-yin Deficiency after Acute Cerebral Infarction | Not meeting study aim |
| 2015 | Zou ZH | Clinical curative effect comparison of“connecting and regulating Ｒen Du” acupuncture versus the traditional acupuncture in treatment of post-stroke insomnia | Not meeting study aim |
| 2015 | Jiao JC | Clinical Observation on Traditional Chinese Medicine and Acupuncture in Treatment of Insomnia | No Data |
| 2015 | Ruan BK | Treating sleep disorders after stroke by acupuncture and drug therapy | No Data |
| 2015 | Zhang ZS | Clinical Study of Acupoint Catgut Embedding for Insomnia During Post-Stroke Recovery | No Data |
| 2016 | Ding BC | Clinical Observation of 30 Cases of Post-Stroke Insomnia Treated with Acupuncture at Dong's Extra Points | Not meeting study aim |
| 2016 | Gu ZL | Clinical Research of Shu Mu Acupoints Acupuncture on TheTreatment of Yin-deficiency and Fire-hyperactivity Type ofPost-stroke insomnia | Not meeting study aim |
| 2016 | Li MY | Therapeutic Observation on Acupuncture for Post——Stroke Insomnia by Tongluo Anshen Needling Therapy | Not meeting study aim |
| 2016 | Liang XL | Effect of Auricular Point Pressing Therapy on Sleep Quality and Neurological Deficits in Patients with Post-Stroke Sleep Disorders | Not meeting study aim |
| 2016 | Liu XY | The Study of the treatment of insomnia After Stroke withDingshen-Needle combine Abdominal Acupuncture Therapy | Not meeting study aim |
| 2016 | Tan YJ | The Clinical Study On Tong Du Tiao Shenacupuncture For The Treatment Ofinsomnia After Stroke | Not meeting study aim |
| 2016 | Fu L | Clinical Efficacy Observation of Couci (Bidirectional Needling) for Post-Stroke Insomnia with Depression | No Data |
| 2016 | Jin FW | Clinical Efficacy Observation of Acupuncture-Moxibustion for Post-Stroke Insomnia (Tanre Raoxin Syndrome) in 30 Cases | No Data |
| 2016 | Wang XH | Clinical observation of Tiaoshen － limian acupuncture combined with estazolam on the treatment of insomnia after cerebral infarction | No Data |
| 2016 | Xu Q | Therapeutic Observation of Scalp Acupuncture plus Thread—embedding at Back-Shu Points for Insomnia in Remission Phase of Stroke | No Data |
| 2017 | Lin YJ | Efficacy Observation of Dispelling Wind-Phlegm & Activating Blood-Collateral Therapy Combined with Auricular Bean Pressing for Post-Apoplexy Insomnia (Fengtan Shangrao Jia Yu Syndrome) | Not meeting study aim |
| 2017 | Lin W | The Clinicalobservation Of The Effect Onsleepdisorder After Apoplexy Byacupuncturing 13 Ghost | Not meeting study aim |
| 2017 | Wang J | Clinical Study On The Treatment Of Insomniaafter Stroke With “Frontal-Three-Needle” | Not meeting study aim |
| 2017 | Wu HL | Efficacy Analysis of Sleep Restriction Therapy Combined with Governor Vessel-Regulating Acupuncture for Post-Stroke Sleep Disorders | Not meeting study aim |
| 2017 | Zhang L | Effect Observation of Acupuncture-Moxibustion Therapy for Post-Stroke Sleep Disorder Patients | Not meeting study aim |
| 2017 | Liu LY | Randomized Controlled Clinical Trials of Eye acupuncture Therapy for Patients with Post-strokeInsomnia | No Data |
| 2017 | Wang N | Clinical Effect Observation of Acupuncture-Moxibustion for Post-Stroke Sleep Disorders | No Data |
| 2017 | Xie S | Clinical observation and analysis of auricular acupoint pressing method for post-stroke depression related insomnia | No Data |
| 2018 | Gai LL | Clinical Observation On Watering To Nourishwood Acupuncture Treatment Of Fire Exuberantdue To Yin Deficiency Type Of Insomia After Stroke | Not meeting study aim |
| 2018 | Li FQ | Effect of Acupuncture at Wuji Points on Sleep Quality of Patients After Cerebral Infarction | Not meeting study aim |
| 2018 | Ma YH | Efficacy Observation of Acupuncture for Post-Stroke Insomnia with Nocturia | Not meeting study aim |
| 2018 | Song YR | “Five heart”acupoints for the treatment ofTCM symptomsofinsomnia after stroke | Not meeting study aim |
| 2018 | Xie XJ | Clinical Efficacy of Acupuncture-Moxibustion for Post-Stroke Sleep Disorders and Its Effect on Neurological Function | Not meeting study aim |
| 2018 | Zhang XL | Clinical effect of integrated traditional Chinese medicine therapy in treatment of insomnia after stroke: an analysis of 35 cases | Not meeting study aim |
| 2018 | Hou ZT | Impacts of the repetitive transcranial acupuncture stimulation on the content of serum orexin A in patients with post-stroke insomnia | No Data |
| 2019 | Guo QF | Clinical Study on Post-stroke Sleep Disorders Treated by Xingnaokaiqiao Acupuncture combined with Yangxuerougan Acupuncture | Not meeting study aim |
| 2019 | Huang X | Clinical Observation on Treatment of Post － stroke Insomnia by Acupuncture and Moxibustion of Invigorating Spleen and Ｒegulating Spirit | Not meeting study aim |
| 2019 | Liang J | Clinical Study On The Treatment Of Insomniaafter Cerebral Hemorrhage With Awnneedle Through Thron Points Of The Dumeridian Group | Not meeting study aim |
| 2019 | Sun QH | Clinical Observation On The Treatment Of Poststroke Cognitive Impairment With Insomnia Byscalp Acupuncture Onacupoints Of The Duand Bladder Meridians | Not meeting study aim |
| 2019 | Zhang XF | Observation and Evaluation of Traditional Chinese Acupuncture-Moxibustion Therapy for Post-Stroke Sleep Disorders | Not meeting study aim |
| 2019 | Ding ZH | Effect Observation of Mind-Regulating Acupuncture on Blood Pressure and Sleep Quality in Stroke Patients | No Data |
| 2019 | Li R | Clinical Observation on the Improvement of Daytime Function of Cerebralinfarction-Relatedinsomnia Patients Byex-Hn1 Ea | No Data |
| 2019 | Li Y | Clinical Observation on the Improvement of cognitive Function in Patients with Sleep Disorder after Cerebral Infarction by Electroacupuncture of Ex-Hn1 | No Data |
| 2019 | Luo X | Clinical Observation of Intradermal Needle Therapy for Post-Stroke Insomnia in 30 Cases | No Data |
| 2019 | Tang HY | Nursing Experience with Auricular Seed Pressing Intervention for Post-Stroke Insomnia | No Data |
| 2019 | Wang WH | Observation of Acupuncture-Moxibustion Efficacy and Neurological Effects in Post-Stroke Sleep Disorders | No Data |
| 2019 | Yang X | Clinical Efficacy of Acupuncture-Moxibustion for Post-Stroke Sleep Disorders and Its Impact on Neurological Function | No Data |
| 2019 | Yuan XH | Clinical Observation of Electroacupuncturesishencong Regulating Sleep Structure in Patients with Cerebral Infarction | No Data |
| 2020 | He SL | Clinical Efficacy of Compound Reinforcing and Relieving Acupuncture Combined with Jin’s Three Needling in Treating Post － Stroke Sleep Disorders and Its Influence to the Scores of PSQI and NIHSS | Not meeting study aim |
| 2020 | Jiang LH | The Efficacy Observation of Tongluo Anshen Acupuncture in Treating Post-Stroke Sleep Disorders | Not meeting study aim |
| 2020 | Liang FN | Therapeutic Effect Ofelectronic Needletwister Oninsomnia After Liver Depression Andfire Type Stroke | Not meeting study aim |
| 2020 | Luo YY | Clinical Observation on Eye Acupuncture Combined with Body Acupuncture for Patients with Post-stroke Insomnia | Not meeting study aim |
| 2020 | Wang JL | A study on treating sleep disorder after stroke by acupuncture | Not meeting study aim |
| 2020 | Wang XX | Treatment of Insomnia after Apoplexy with "Sanshen Acupuncture" | Not meeting study aim |
| 2020 | Wang ZH | Clinical Study of Acupuncture Combined with Diazepam Tablets for Post-Stroke Insomnia | Not meeting study aim |
| 2020 | Ye GX | Study on Clinical Efficacy Evaluation of“Restoring Consciousness and Regulating Fu-organs”Acupuncture Treating Insomnia after Stroke | Not meeting study aim |
| 2020 | Zhang JX | Clinical Observation on Combining Yuan-Luo Acupoint ofMeridian-Connection Therapy for Insomnia after Stroke due toImbalance between Heart and Kidney | Not meeting study aim |
| 2020 | Zhang XY | Clinical observation on acupuncturing ‘Wuxin acupoints’ in treating insomnia after stroke with disharmony between heart and kidney | Not meeting study aim |
| 2020 | Zhang XX | Effect of Auricular Magnetic Bead Therapy and KaitianmenCombined Therapyon Sleep and Fatigue ofThe Patients AfterStroke in The Community | Not meeting study aim |
| 2020 | Zhu QW | Clinical effect observation of acupuncture method of "Xia0Xing Nao" combined with cervical point in treating insomniaafter stroke | Not meeting study aim |
| 2020 | Fan J | Effects of"Deep and long stay" acupuncture on sleep, earlymorning and before sleep blood pressure in stroke patientswith insomnia and hypertension | No Data |
| 2020 | Su FY | Observation on the Rehabilitation Effect of Acupuncture and Drug Therapy on Patients with Ischemic Stroke Hemiplegia Complicated with Sleep Disorder | No Data |
| 2020 | Wang Q | Evaluation of Acupuncture-Moxibustion Efficacy and Neurological Impact in Post-Stroke Sleep Disorders | No Data |
| 2020 | Wu YC | Efficacy Observation of Conventional Acupuncture Combined with Press Needles for Post-Stroke Insomnia Patients | Not meeting study aim |
| 2021 | Shi HX | Clinical Observation on the Therapeutic Effect of Auricular Magnetic Therapy in Patients with Post-Stroke Sleep Disorders | Not meeting study aim |
| 2021 | Hong BQ | Clinical observation of regulating spirit acupuncture techniquecombined with drugs in treatment of post-stroke cireadiam rhythm sleep-wake disorder | Not meeting study aim |
| 2021 | Peng YX | Randomized Controlled Study of Acupuncture for Regulating Triple Energizer Qi on Improvement of Sleep and Quality of Life in Post-stroke Insomnia Patients with Deficiency of Heart and Spleen Type | Not meeting study aim |
| 2021 | Song SL | Effect of Auricular Point Pressing Combined with Acupuncture on Sleep Quality and Neurotransmitters in Post-Stroke Sleep Disorder Patients | Not meeting study aim |
| 2021 | Wu JL | Analysis of the therapeutic effect of "Jin Sanzhen" Tiaoshen Acupuncture on sleep disorders in elderly patients with post-stroke depression | Not meeting study aim |
| 2021 | Wu QH | Clinical Observation Of Sun'S Abdominalacupuncture Therapy On Treatmentof Insomnia After Stroke | Not meeting study aim |
| 2021 | Yang LX | Clinical Observation on Tongyuan Acupuncture Treatment Methods for Post-stroke sleep disorders | Not meeting study aim |
| 2021 | Yu R | The Clinical Research Of Therapy Forstroke-Related Sleep Disorders Wite The Tongdu Zhi Yu Acupunature Of The Therapeuticeffect And The Effects Of S-Ht、Da Levels | Not meeting study aim |
| 2021 | Zhang JS | Efficacy study of acupuncture in improving depressive state in post-stroke depression patients | Not meeting study aim |
| 2021 | Zhang ZM | Effect of Ear Buried Beans on Hemiplegia Exercise and Sleep of Ischemic Stroke | Not meeting study aim |
| 2021 | Zhuo YW | The Effect study of Meridian Flow Timing AuricularPoint Magnetic Bead Plaster Therapy on Patients WithSleep Disorders After Ischemic Stroke | Not meeting study aim |
| 2021 | Wu X | Nursing Analysis of Auricular Seed Pressing Intervention for Post-Stroke Insomnia | No Data |
| 2021 | Shao B | Effect of acupuncture on the daytime function in patients with post-stroke sleep-wake disturbance: a randomized controlled trial | No Data |
| 2021 | Jiang DL | Clinical Observation on the Effect of Cizhu Ear AcupointPressing on Insomnia of Heart and Kidney Disjoint afterIschemic Stroke | Not meeting study aim |
| 2022 | Li CY | Clinical study of heart－brain co－therapy acupuncture in treating sleep rhythm disorders of ischemic stroke | Not meeting study aim |
| 2022 | Li JJ | Clinical Observation on Liver and Kidney Maintenance Acupuncture Combined with Oryzanol in Treatment of Insomnia after Ischemic Stroke | Not meeting study aim |
| 2022 | Li XH | Clinical Observation on Nazi Method of Midnight-Noon inTreating the Post-Stroke Insomnia (deficiency of heart and spleen) | Not meeting study aim |
| 2022 | Lian J | The Effect of Auricular Point Sticking Therapy in Treating Post-stroke Insomnia | Not meeting study aim |
| 2022 | Song Y | Clinical Observation on the Treatment of Post-strokeSleep Inversions by Ren Du Trans-acupuncture | Not meeting study aim |
| 2022 | Yang J | Effect ofAuricular Point Embedding Beans Combined with Acupuncture on Sleep Quality and Swallowing Function ofPatients with Cerebral Infarction | Not meeting study aim |
| 2022 | Xie H | Clinical observation of Tiaoqi Guben Acupuncture in theTreatment of Heart and Spleen Deficiency Insomnia afterIschemic Stroke | Not meeting study aim |
| 2022 | Zhao Q | Effect of Tiaoshen Qianyang acupuncture on blood pressure and sleep quality in patients with stroke-related sleep disorders complicated with hypertension | Not meeting study aim |
| 2022 | Guo Y | Efficacy Analysis of Auricular Point Pressing for Insomnia in Post-Stroke Recovery Patients | No Data |
| 2022 | Lu HJ | Effect Observation of Midday-Midnight Ebb-Flow Auricular Press-Needle as Adjuvant Therapy for Post-Stroke Insomnia (Tanre Raoxin Syndrome) | No Data |
| 2022 | Su GL | Therapeutic Effect of Meridian Pulse Acupuncture in Rehabilitation for Post-Stroke Sleep Disorder Patients | No Data |
| 2023 | Chi SJ | Clinical Observation on the Treatment of Sleep Deviation after Stroke by Acupuncture from ' Heart and Gallbladder | Not meeting study aim |
| 2023 | Guo D | Effects of Acupuncture Combined with Auricular Acupoint Pressing with Bean in Treatment of Insomnia after Ischemic Stroke Based on Midnight － Noon Ebb － Flow Theory | Not meeting study aim |
| 2023 | Jin XX | Clinical Efficacy of Jiannao Anshen Acupuncture in Post-Stroke Insomnia Patients | Not meeting study aim |
| 2023 | Li D | Efficacy observation of HE’s San Tong acupuncture method for post-stroke sleep wake disorders and the impact on sleep quality | Not meeting study aim |
| 2023 | Li SL | Observation on the clinical efficacy of cheeks acupuncturein the treatment ofinsomnia after stroke | Not meeting study aim |
| 2023 | Li YC | Clinical Observation on the Treatment of Insomnia after Strokeby Tongluo Anshen Acupuncture | Not meeting study aim |
| 2023 | Lin L | Clinical Observation of Abdominal Acupuneture on Post-stroke Sleep Disorders : A Randomized Controlled Trial | Not meeting study aim |
| 2023 | Song CH | Clinical Study of ＲN14 Triple Needling Combined with Umbilical Acupuncture in Treatment of Post － Stroke Insomnia | Not meeting study aim |
| 2023 | Tan XY | Clinical Efficacy Analysis of Meridian Pulse Acupuncture in Rehabilitation for Post-Apoplexy Sleep Disorder Patients | Not meeting study aim |
| 2023 | Xue TY | Clinical efficacy observation of wrist-ankle acupuncturecombined with Tiaoshen Tongluo acupuncture in the treatmentofInsomnia after stroke(liver-fire disturbing heart type) | Not meeting study aim |
| 2023 | Yu X | Efficacy observation of the unblocking-regulating acupuncture method for post-stroke insomnia | Not meeting study aim |
| 2023 | Zou C | Clinical Study of Governor Vessel-Jiaotai Acupuncture for Post-Stroke Insomnia | Not meeting study aim |
| 2024 | Zeng YH | Clinical study of back-shu points and front-mu points combination combined with umbilical acupuncture on circadian rhythm sleep-wake disorder after stroke | Not meeting study aim |
| 2024 | Gao F | Auricular Acupressure Combined with Kaitianmen in the Treatment of Stroke with Insomnia | Not meeting study aim |
| 2024 | Gao YH | Effects of acupuncture therapy on sleep disorders and neuroimmunological indexes after stroke | Not meeting study aim |
| 2024 | Huang CM | Clinical observation on post-stroke sleep disorders with pattern of phlegm-heat disturbing the heart treated by Lingnan scalp acupuncture | Not meeting study aim |
| 2024 | Huang Y | The Impaet of Acupuneture Therapy on Neurological Impairmentand Sleep Quality in Patients with Sleep Disorders after Stroke | Not meeting study aim |
| 2024 | Li LR | The Clinical Effect of Ear Manipulation Combined with Ear Acupoint Pressing Therapy on Sleep Disorders in Stroke Patients | Not meeting study aim |
| 2024 | Li F | Clinical Efficacy Observation of Midday-Midnight Ebb-Flow (Na Zi Method) Combined with Conception-Governor Vessel Circulation Acupuncture for Post-Stroke Insomnia | Not meeting study aim |
| 2024 | Wang DQ | Clinical Study of Huangdi Neizhen for Post-Stroke Insomnia | Not meeting study aim |
| 2024 | Ma QH | Observation on the Efficacy of ZiwuliuzhuAcupuncture Method in the Treatment of Post-Stroke(Wind-Phlegm Obstruction Type) Sleep Disorders | Not meeting study aim |
| 2024 | Yu D | Clinical study on treating post-stroke insomnia by acupuncturemethod of“Zhenjing Anshen’ | Not meeting study aim |
| 2024 | Han M | Clinical Study of Abdominal Acupuncture for Post-Stroke Sleep Disorders | Not meeting study aim |
| 2024 | Song RY | Warm acupuncture and moxibustion for treating post－stroke sleep disorders of phlegm-heat disturbing the heart type | No Data |
| 2024 | He RF | Therapeutic Effect of Warm Needle Moxibustion at Sanyinjiao (SP6) Combined with Auricular Seed Pressing for Geriatric Post-Stroke Insomnia | Not meeting study aim |
